# Combined nanometric and phylogenetic analysis of unique endocytic compartments in *Giardia lamblia* sheds light on the evolution of endocytosis in Metamonada

**DOI:** 10.1186/s12915-022-01402-3

**Published:** 2022-09-21

**Authors:** Rui Santos, Ásgeir Ástvaldsson, Shweta V. Pipaliya, Jon Paulin Zumthor, Joel B. Dacks, Staffan Svärd, Adrian B. Hehl, Carmen Faso

**Affiliations:** 1 Institute of Parasitology, University of Zürich, Winterthurerstrasse 266a, 8057 Zürich, Switzerland; 2 Institute of Anatomy, University of Zürich, Winterthurerstrasse 190, 8057 Zürich, Switzerland; 3 Department of Cell and Molecular Biology, University of Uppsala, Husargatan 3, 752 37 Uppsala, Sweden; 4 Department of Microbiology, National Veterinary Institute, 751 23 Uppsala, Sweden; 5 Division of Infectious Diseases, Department of Medicine, University of Alberta, Edmonton, Alberta Canada; 6 School of Life Sciences, École Polytechnique Fédérale de Lausanne, Lausanne, Switzerland and Swiss Institute of Bioinformatics, Lausanne, Switzerland; 7 Amt für Lebensmittelsicherheit und Tiergesundheit Graubünden, Chur, Switzerland; 8 Institute of Parasitology, Biology Centre, CAS, v.v.i., Branisovska 31, 370 05 Ceske Budejovice, Czech Republic; 9 Institute of Cell Biology, University of Bern, Bern, Switzerland; 10 Multidisciplinary Center for Infectious Diseases, Vetsuisse, University of Bern, Bern, Switzerland

**Keywords:** Endocytosis, Super-resolution microscopy (SRM), Volumetric electron microscopy, Peripheral endocytic compartments (PECs), Convergent evolution, Metamonada, *Giardia*, *Tritrichomonas*, *Spironucleus*, Peripheral vacuoles

## Abstract

**Background:**

*Giardia lamblia*, a parasitic protist of the Metamonada supergroup, has evolved one of the most diverged endocytic compartment systems investigated so far. Peripheral endocytic compartments, currently known as peripheral vesicles or vacuoles (PVs), perform bulk uptake of fluid phase material which is then digested and sorted either to the cell cytosol or back to the extracellular space.

**Results:**

Here, we present a quantitative morphological characterization of these organelles using volumetric electron microscopy and super-resolution microscopy (SRM). We defined a morphological classification for the heterogenous population of PVs and performed a comparative analysis of PVs and endosome-like organelles in representatives of phylogenetically related taxa, *Spironucleus* spp. and *Tritrichomonas foetus*. To investigate the as-yet insufficiently understood connection between PVs and clathrin assemblies in *G. lamblia*, we further performed an in-depth search for two key elements of the endocytic machinery, clathrin heavy chain (CHC) and clathrin light chain (CLC), across different lineages in Metamonada. Our data point to the loss of a bona fide CLC in the last Fornicata common ancestor (LFCA) with the emergence of a protein analogous to CLC (*Gl*ACLC) in the *Giardia* genus. Finally, the location of clathrin in the various compartments was quantified.

**Conclusions:**

Taken together, this provides the first comprehensive nanometric view of *Giardia*’s endocytic system architecture and sheds light on the evolution of *Gl*ACLC analogues in the Fornicata supergroup and, specific to Giardia, as a possible adaptation to the formation and maintenance of stable clathrin assemblies at PVs.

**Supplementary Information:**

The online version contains supplementary material available at 10.1186/s12915-022-01402-3.

## Background

Endomembrane compartments, while present in a few prokaryotic lineages [1], have evolved and greatly diversified across eukaryotic lineages. A fundamental task performed by some membrane-bounded organelles is endocytosis—the controlled and directed uptake of nutrients and other materials from the extracellular space into the cell by membrane transport. Fluid phase or receptor-bound material at the cell surface is internalized via invaginations and formation of vesicles at the plasma membrane, mediated by clathrin-coated vesicles (CCVs) [2, 3]. In turn, CCVs fuse with early endosomes which mature into late endosomes upon lysosome fusion [4, 5]. Clathrin coats are also involved in protein secretion forming exocytic transport vesicles derived from the trans-Golgi compartment and play a role in Golgi apparatus reassembly after mitotic cell division [6, 7].

Evolutionary adaptations of endocytic pathways to specific environmental niches and nutrient sources are especially relevant to species adopting a fully parasitic or commensal lifestyle [8–11]. Within the extant Metamonada supergroup [12–14], the parasitic protist *Giardia lamblia* (syn.: *intestinalis* or *duodenalis*) evolved a distinct endocytic pathway, which reflects its adaptation to the host intestinal lumen environment. This unicellular parasite is responsible for > 300 million cases annually of water-borne infections causing gastroenteritis—giardiasis—with a higher incidence in low- to middle-income countries [15]. *Giardia* is the etiological agent for symptomatic gastroenteritis in 15% of children in developing countries, with 1–2% fatality in children with severely compromised health status [16, 17]. There is a strong association of *Giardia* infections with chronic conditions such as irritable bowel syndrome or inflammatory bowel disease as a result of intestinal barrier function disruption and microbiome dysregulation [18, 19].

The cellular evolution of the *Giardia* genus as an obligate parasite adapted to the small intestinal niche of vertebrates is characterized by a reduction in subcellular compartment diversity. Peroxisomes, late endosomes and a permanent stacked Golgi complex have not been detected in *Giardia* [20]. Two nuclei [21], an extensive endoplasmic reticulum (ER) [22], highly reduced mitochondria-derived organelles—the mitosomes [23]—and peripheral vesicles (PVs) [24] are the only membrane-bounded organelles with conserved morphology and function documented in the *Giardia* trophozoite [25–27].

The complex array of PV organelles as the only documented endocytic membrane compartment system in *Giardia* is responsible for the uptake of fluid-phase and membrane-bound material [27–29]. These organelles acidify and presumably serve as digestive compartments with the capability for sorting after processing, similar to early and late endosomes and lysosomes [24]. The static system of PV organelles [27, 30] is mainly restricted to the peripheral cortex below the plasma membrane (PM) of the *Giardia* trophozoite dorsal side. PV morphology was investigated using high-resolution electron microscopy serial sectioning and three-dimensional reconstruction [27]. These organelles were resolved as tubular structures in close proximity to funnel-shaped invaginations of the PM [27]. Our current working model for bulk fluid-phase uptake of extracellular material into PVs invokes a “kiss and flush” mechanism, whereby acidified PV membranes and the PM transiently form channels at invaginations allowing exchange between PV lumen content and the extracellular space at regular intervals. Endocytosed material is digested and transported towards the cell interior while residual material and waste are flushed to the extracellular space in the next round of membrane fusion, thus completing the PV cycle [26, 27]. The characterized molecular complement of the PV surface includes ESCRT components [11] and focal long-lived albeit non-vesicular accumulations of clathrin heavy chain (CHC) molecules and their main interactors, collectively termed clathrin assemblies [27]. A notable member of these is a putative *Giardia* clathrin light chain *Gl*CLC (ORF 4259), a scarcely annotated protein presenting strong interaction with *Gl*CHC, similar dynamics as measured by FRAP, matching sub-cellular localization and considerable (*ca*. 50%) overlap of its predicted 3D structure compared to the corresponding prediction for well-characterized CLCs [27]. The function of these stable focal assemblies, as well as additional components at the interface of the PV membranes and the PM, has proved elusive [27]. However, a transient association of several members of the family of adaptor proteins (AP) suggests a role in dynamic processes linked to the uptake of fluid-phase and receptor-bound material into PVs [26, 27].

In this report, we address open questions concerning *G. lamblia*’s PV ultrastructure and its associated molecular machinery in a comparative approach with one closely and one more distantly related fornicata and metamonada species, *Spironucleus* sp. and *Tritrichomonas foetu*s, respectively. Using volumetric electron microscopy and super-resolution light microscopy, we developed a classification of PVs based on organelle morphology. Comparative analysis of *Giardia*’s PVs with endocytic compartments of fornicata and metamonada species emphasized the genus-specific nature of the *Giardia* endocytic system architecture. In addition, using a combination of co-immunoprecipitation and phylogeny techniques, we provide evidence that the putative clathrin light chain *Gl*CLC [27] is unique to the *Giardia* genus and evolved de novo as structurally analogous to CLC after the loss of *a bona fide* CLC in the last Fornicata common ancestor (LFCA). Taken together, the emergence of a unique and highly polymorphic endocytic system such as the one found in the genus *Giardia* is linked to the proposed convergent evolution of an independent CLC analogue concomitant with the loss of a mostly conserved CLC orthologue.

## Results

### Complete FIB-SEM rendering of a *G. lamblia* trophozoite reveals a novel landscape of vesicular compartments

**Fig. 1:**
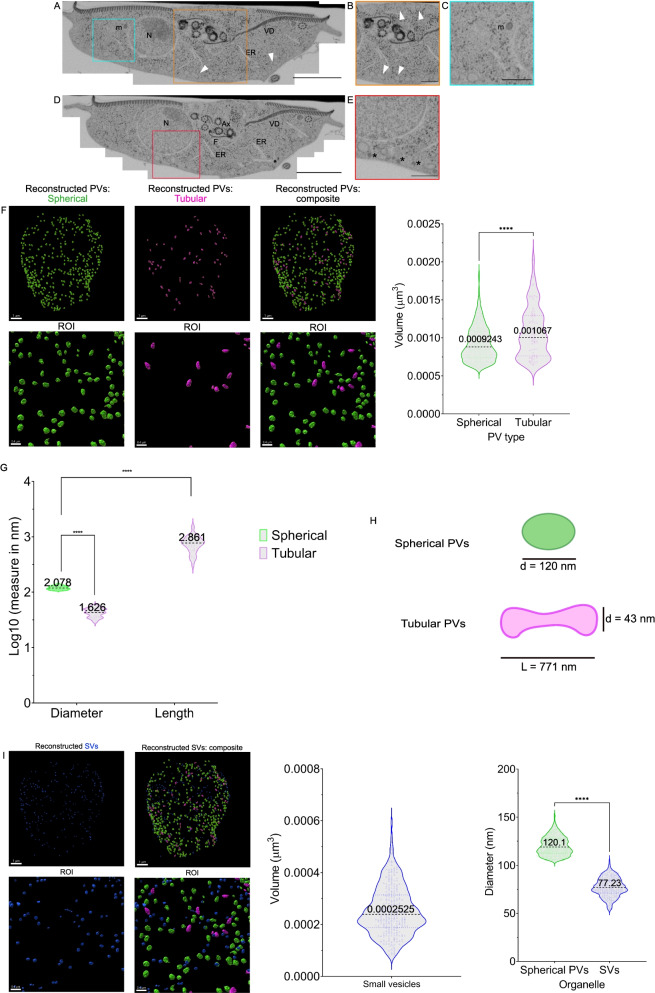
Complete scanning of a *Giardia* trophozoite by focused ion beam scanning electron microscopy (FIB-SEM). **A**, **D** A whole *G. lamblia* trophozoite was scanned with an isotropic resolution of 5 nm showing the nucleus (N), the endoplasmic reticulum (ER), cytoskeletal features such as the ventral disc (VD), funis (F) and axonemes (Ax) and mitosomes (m). Peripheral vesicles (PV) are marked by arrowheads. Smaller, electron-dense vesicles (small vesicles (SV)) are also documented (asterisks). **B** Region of interest of **A** highlighting PVs of different morphology (arrowheads). **C** Mitosomes proximal to ER membrane. **E** Region of interest of **D** highlighting SVs (asterisks). **F** Full reconstruction of PVs with ilastik and rendering in Imaris showing at least two PV morphologies: spherical (green) and tubular (violet). Violin plot: 403 spherical and 64 tubular PVs were segmented out. Spherical PVs average a volume of 0.0009243 ± 0.0003322 μm^3^ with a 95% confidence interval between 9.022 × 10^−4^ and 9.464 × 10^−4^ μm^3^. Tubular PVs average a volume of 0.001067 ± 0.0003322 μm^3^ with a 95% confidence interval between 0.0009843 and 0.001150 μm^3^. **G** Tubular PV lengths are larger than their diameter (*p*-value < 0.0001) and also larger than spherical PVs (*p*-value < 0.0001) (values presented in log_10_ form). **H** Diameters of spherical PVs average 120.1 ± 9.507 μm (95% CI: [119.2; 121.0]). Tubular PV diameters average 43.01 ± 7.816 μm (95% CI: [41.06; 44.96]). Tubular PV lengths average 771.3 ± 266.2 μm (95% CI: [704.8; 837.8]). **I** Reconstruction of 269 SVs averages volume at 0.0002525 ± 9.280 × 10^-5^ μm^3^ with a 95% confidence interval between 0.0002414 and 0.0002637 μm^3^ (left violin plot). This equals an average diameter of 77.23 ± 9.666 nm with a 95% confidence interval between 76.07 and 78.40 nm. Spherical PV diameter averages 120.1 ± 9.507 nm with a 95% confidence interval between 119.2 and 121 nm (right violin plot). The difference in the diameter between SVs and spherical PVs is statistically significant (****p-value < 0.0001, *t*-Student significance test). Scale bars: **A**, **D** 2 μm; **B**, **C** and **E** 500 nm. ROI, region of interest

Volumetric scanning electron microscopy (vSEM) is currently considered the gold standard for the determination of biological ultrastructure [31]. One type of vSEM is focused ion beam electron scanning microscopy (FIB-SEM) where a beam of gallium ions is used to mill and image consecutive layers of an embedded biological sample, resulting in a voxel resolution as low as 1–2 nm [32]. This technique allows for sectioning and imaging of entire cells [33] and was previously implemented for the partial rendering of *G. lamblia* trophozoite sections [27, 34]. Here, we sectioned for the first time a complete *G. lamblia* trophozoite at a voxel resolution of 125 nm^3^ (5 × 5 × 5 nm) after high-pressure freezing (HPF) and embedding. Images representing the sagittal plane adjacent to the cell centre (Fig. 1A, D and Additional file 1: Fig. S1A and S1B) show all the major cell compartments such as the nuclei (Fig. 1A, N), the endoplasmic reticulum (Fig. 1A, ER), mitosomes (Fig. 1A, C, m) and elements of the cytoskeleton: axonemes (Ax), funis (F) and the ventral disc (VD) (Fig. 1D [35];). Two different types of small cytoplasmic organelles are observed: PVs (arrow heads) with heterogenous morphology and smaller and electron-dense membrane vesicles of uniform size and appearance which we termed small vesicles (SVs; asterisks) (Fig. 1B, E).

After serial sectioning and alignment with TrakEM [36], we used the supervised machine learning (ML) tool ilastik for pixel-based image segmentation of PVs and SVs [37, 38]. The algorithm collection performs supervised learning and recognition of patterns based on ground truth training provided by the user. Patterns are sorted into classes. Once the algorithm is trained on a subset of image data, it is used to analyse complete datasets and assign features to different classes following a decision tree method [39, 40]. This process enabled the three-dimensional rendering of selected trophozoite features: the complete cytoskeleton, the ER, PVs and mitosomes (Additional file 1: Fig. S1C). In addition, we were able to calculate the volume of the cell at 138 μm^3^ as well as the average volume of mitosome organelles (*N* = 14) at 0.001093 ± 0.0005698μm^3^ with a 95% confidence interval between 0.0007643 and 0.001422 μm^3^ (Additional file 1: Fig. S1D).

Similarly, supervised ML-assisted pixel segmentation and object clustering analysis allowed the identification of two statistically distinct morphological classes of PVs: spherical and tubular/elongated PVs. Individual PV organelles of both classes (*N* = 467) were rendered in three dimensions (Fig. 1F and Additional file 2: Video S1). Spherical PVs average a volume of 9.243 × 10^−4^ ± 3.322 × 10^−4^ μm^3^ with a 95% confidence interval between 9.022 × 10^−4^ and 9.464 × 10^−4^ μm^3^ while tubular PVs average a volume of 1.067 × 10^−3^ ± 3322 × 10^−4^ μm^3^ with a 95% confidence interval between 9.843 × 10^−4^ and 1.150 × 10^−3^ μm^3^, a statistically significant difference (*t*-Student test, *p* < 0.0001), corroborating PV grouping in these two classes. We also determined the length of tubular PVs to be significantly larger than their diameter and also larger than the diameter of spherical PVs (Fig. 1G). Spherical PVs average a diameter of 120.1 ± 9.507 μm (95% CI: [119.2; 121.0]). The diameter of tubular PVs is calculated to average 43.01 ± 7.816 μm (95% CI: [41.06; 44.96]). Tubular PVs average a length of 771.3 ± 266.2 μm (95% CI: [704.8; 837.8]) (Fig. 1H).

To further investigate the morphological heterogeneity of PVs, we analysed trophozoite ultrastructure using freeze-fracture scanning electron microscopy. We documented PV heterogeneity and the presence of spherical and tubular PV forms (Additional file 3: Fig. S2). Additional ultrastructural studies using transmission electron microscopy (TEM) were consistent with this classification (Additional file 4: Fig. S3A and B).

We proceeded with the rendering of 269 SVs—small spherical vesicles, with distinctly higher electron density than PVs and what could be a coat on the cytoplasmic side of the delimiting membrane (Fig. 1I). SVs were also identified by TEM (Additional file 4: Fig. S3), proximal to the PM. SVs average a volume of 2.525 × 10^−4^ ± 9.280 × 10^−5^ μm^3^ with a 95% confidence interval between 2.414 × 10^−4^ and 2.637 × 10^−4^ μm^3^ (Fig. 1I, left violin plot). This equals an average diameter of 77.23 ± 9.666 nm with a 95% confidence interval between 76.07 and 78.40 nm, differing significantly from spherical PVs which average 120.1 ± 9.507 nm with a 95% confidence interval between 119.2 and 121 nm (*p* < 0.0001) (Fig. 1I, right violin plot). Thus, there is statistical support for SVs as a distinct category of membrane-bounded vesicles (Additional file 4: Fig. S3A and S3C).

Taken together, these findings lead us to hypothesize that, unlike previously thought, PVs are morphologically heterogenous and may comprise different functional categories [41–43]. However, these data are currently insufficient to determine whether distinct morphologies correlate with distinct functions.

### Combining super-resolution microscopy with ML-assisted image analysis identifies three classes of endocytic compartments in *G. lamblia* trophozoites

FIB-SEM as a technique is not well-suited to the investigation of large cell numbers, and TEM cannot readily provide 3D volumetric information on subcellular compartments. Hence, to address PV heterogeneity in more detail, we continued our investigation of *Giardia* endocytic compartments by super-resolution light microscopy (SRM) techniques and ML-assisted image analysis of compartment shapes.

**Fig. 2:**
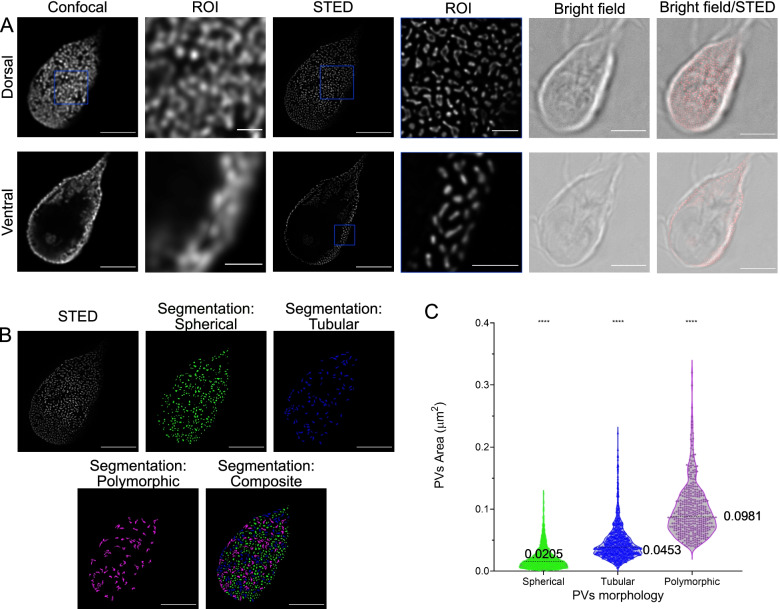
Super-resolution imaging of *Giardia lamblia* peripheral vesicles with stimulated emission depletion (STED). **A** *Giardia* trophozoites loaded with 10-kDa Dextran-AlexaFluor 594 were imaged using confocal and STED microscopy. Dorsal (upper row) and ventral (lower row) regions are represented. In contrast to confocal imaging, STED microscopy allows to separate individual organelles and to visualize different endocytic compartment morphologies. ROI, region of interest. **B** Organelle segmentation with ilastik distinguishes three dextran-labelled PV categories. **C** PV areas were calculated post-segmentation on maximum projections of the dorsal regions of 15 cells, using ilastik. Spherical PVs (green, *N* = 1684) have an average area of 0.0205 ± 0.0169 μm^2^ with a 95% confidence interval between 0.0197 and 0.0213, tubular PVs (blue, *N* = 835) have an average area of 0.0453 ± 0.0278 μm^2^ with a 95% confidence interval between 0.0435 and 0.0472 and polymorphic PVs (magenta, *N* = 400) have an average area of 0.0981 ± 0.0429 μm^2^ with a 95% confidence interval between 0.0939 and 0.102. The differences in the area are statistically significant (ANOVA; *p*-value < 0.0001). Scale bars: **A** 5 μm and 1 μm (ROIs), **B** 5 μm

The dimensions of *Giardia* endocytic compartments are well below the diffraction limit of conventional light microscopy [44]. To overcome the Abbe diffraction barrier, we used stimulated emission depletion microscopy (STED), potentially achieving a lateral (*x*, *y*) and axial (*z*) resolution of 25–50 nm and 60–100 nm, respectively. This technique overall decreases the point spread function signal from the illuminated region [45, 46] and allows for accurate imaging of trophozoite PV lumina loaded with a highly photostable fluid phase marker (10-kDa Dextran-Alexa Fluor 594) which is readily taken up into PVs via the fluid phase endocytic pathway (Fig. 2A and Additional file 5: Video S2). In addition to spherical and tubular PVs documented in FIB-SEM, using STED, we also determined the presence of polymorphic dextran-labelled organelles, i.e. spherical PVs with elongated rods (Fig. 2A, representative image of *N* = 15 cells). All labelled PVs were further analysed using the ML-assisted algorithm of the ilastik program suite. We first performed a supervised pixel segmentation followed by a supervised object classification. In this second step, we defined and trained the classifier in three organelle morphologies: spherical, tubular and polymorphic. The latter comprised characteristics of both vesicular and tubular classes, generally with spherical centres with attached tubular protrusions (Fig. 2B). After organelle classification, we measured their projected areas. Spherical organelles (*N* = 1684) have an average projected area of 0.0205 ± 0.0169 μm^2^ with a 95% confidence interval between 0.0197 and 0.0213 μm^2^. Tubular endocytic organelles (*N* = 835) present an average projected area of 0.0453 ± 0.0278 μm^2^ with a 95% confidence interval between 0.0435 and 0.0472 μm^2^. Polymorphic organelles (*N* = 400) have an average projected area of 0.0981 ± 0.0429 μm^2^ with a 95% confidence interval between 0.0939 and 0.102 μm^2^. ANOVA analysis reveals that each of the three categories is indeed significantly distinct (*p* < 0.0001) based on the projected surface area (Fig. 2C). This lends further support to the possibility that PV morphological heterogeneity may have functional implications.

Although a STED microscopy-based approach clearly allows the resolution of individual organelles as small as PVs, the distinctly lower axial resolution remains limiting for the three-dimensional rendering of organelles. Therefore, to push the boundaries of resolution and to further characterize PV morphology, we employed single-molecule localization microscopy (SMLM) [47–49].

**Fig. 3:**
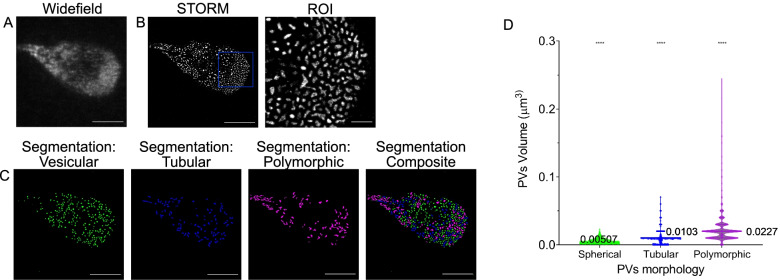
Super-resolution imaging of *Giardia lamblia* PVs by stochastic object reconstruction microscopy (STORM). **A** Widefield microscopy-based imaging of a *Giardia* trophozoite loaded with 10-kDa Dextran-Alexa Fluor 647. **B** Reconstruction of single-molecule events using the Fiji plugin Thunderstorm. As with STED imaging, different PV morphologies are observed. **C** PVs were segmented (*N* = 10 cells) with the help of ilastik, and volumes were calculated and plotted in (**D**). Spherical PVs (green, *N* = 1989) present an average volume of 0.00507 ± 0.00336 μm^3^ with a 95% confidence interval between 0.00492 and 0.00522 μm^3^, tubular PVs (blue, *N* = 838) present an average volume of 0.0103 ± 0.00925 μm^3^ with a 95% confidence interval between 0.00967 and 0.0109 μm^3^ and polymorphic PVs (magenta, *N* = 1494) present an average volume of 0.0227 ± 0.0214 μm^3^ with a 95% confidence interval between 0.0216 and 0.0238 μm^3^. The differences in the area are statistically significant (ANOVA; *p*-value < 0.0001). Based on these and previously shown data, the renaming of PVs to peripheral endocytic compartments (PECs) is proposed. Scale bars: 5 μm and 1 μm (ROI)

*Giardia* PVs in trophozoites were loaded with a 10-kDa Dextran-Alexa Fluor 647 fluid phase marker with a high degree of photostability to survive repeated cycles of photoactivation and excitation in SMLM experiments [50, 51]. After the acquisition, images were reconstructed using the ImageJ plugin ThunderStorm which performs signal centroid calculation, image reconstruction and output [52, 53]. Dextran uptake in PVs was confirmed using conventional widefield microscopy (Fig. 3A). STORM image reconstruction shows the subcellular distribution of the fluorescent marker and defines individual organelle lumina (Fig. 3B, representative image of *N* = 10 cells). A closer inspection revealed the presence of morphologically distinct endocytic organelles as previously observed in our FIB-SEM and STED datasets (Fig. 3B, ROI and Additional file 6: Video S3). We again used the supervised ML-assisted algorithm in ilastik to classify the different morphologies. After a pixel segmentation routine, we performed object classification using supervised ground truth training on subsets of organelle images. Three categories of PVs were defined: spherical, tubular and polymorphic (Fig. 3C). To test whether the morphological categorization was consistent with categorization based on organelle volume, we calculated the average lumina volumes of > 4000 organelles from the three PV categories. ANOVA testing of organelle volumes for vesicular (0.00507 ± 0.00336 μm^3^, *N* = 1989, 95% confidence interval: [0.00492, 0.00522] μm^3^), tubular (0.0103 ± 0.00925 μm^3^, *N* = 838, 95% confidence interval: [0.00967, 0.0109] μm^3^) and polymorphic (0.0227 ± 0.0214 μm^3^, *N* = 1494, 95% confidence interval: [0.0216, 0.0238] μm^3^ organelles confirmed statistically significant (*p* < 0.0001) morphological differences (Fig. 3D and summarized in Additional file 7: Table S1).

Taken together, the data generated using three distinct imaging techniques clearly demonstrate PV heterogeneity which may be linked to distinct functions and/or maturation states in this unique endocytic system. To reflect this novel finding and taking into account that these endocytic and peripherally localized organelles are neither proper vesicles nor canonical vacuoles, we propose renaming PVs to peripheral endocytic compartments (PECs).

### Comparative analysis of endocytic and secretory organelles in *Giardia, Spironucleus* sp. and *T. foetus*

*Giardia* spp. have evolved a unique cell architecture including a dedicated organelle for attachment to the small intestinal epithelium—the ventral disc (VD) [35, 54]. In turn, this innovation defines a distinct dorsal-ventral as well as antero-posterior polarization of the flagellated trophozoite, marked by swimming directionality. PVs/PECs localize exclusively to the dome-shaped dorsal parasite PM except for a small circular patch at the centre of the VD called the bare zone [26, 27]. The result is a maximally decentralized architecture of the *Giardia* endocytic system forming a single-layer interface of what we now appreciate as 3 morphologically distinct organelle classes between the cell exterior and the cytoplasm/ER [27, 30]. We asked whether this type of decentralized sub-PM localization and polymorphic morphology of endocytic compartments was also represented in other tractable, phylogenetically related members of the Diplomonadida as well as more distant metamonada lineages which do not have a VD, i.e. *Spironucleus vortens* and *Spironucleus salmonicida* and the parabasalid *Tritrichomonas foetus.*

**Fig. 4:**
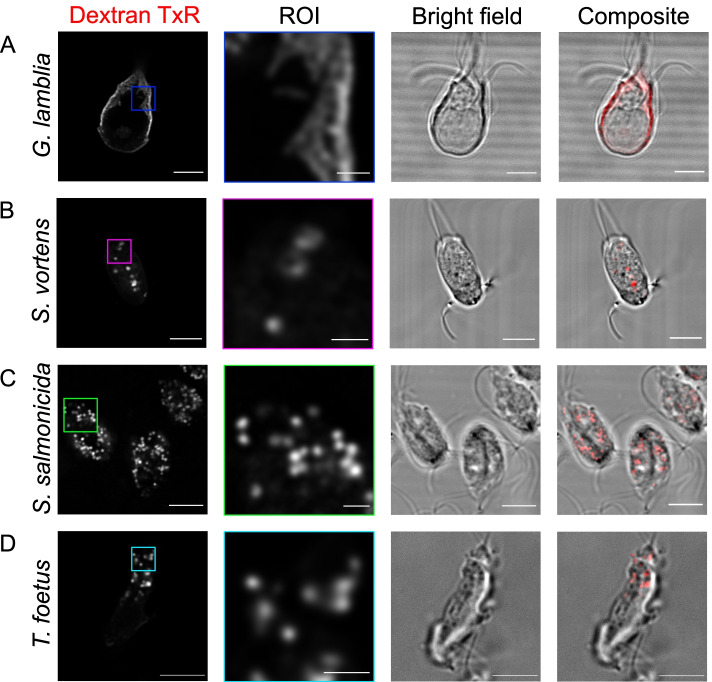
Uptake of fluorescently labelled dextran in metamonada and discoba members: *G. lamblia*, *S. vortens*, *S. salmonicida* and *T. foetus* after 30 min. **A** *G. lamblia* cells present endocytic compartments spread in the cell periphery, unresolved by conventional light microscopy. On the other hand, **B** *S. vortens*, **C** *S. salmonicida* and **D** *T. foetus* present vesicular endocytic compartments. Scale bars: 5 μm (full cells) and 1 μm (ROI)

The diplomonads *S. vortens* and *S.salmonicida* are amongst the closest tractable relatives of *G. lamblia* that can be grown axenically under similar conditions [55–59]. Their endocytic compartments and machineries are partially characterized, with some reports of large vacuolar structures detected by electron microscopy in trophozoites [60, 61]. Unlike *Giardia*, both species lack dorso-ventral polarization but display a distinct antero-posterior axis. Putative endocytic organelles in *S. vortens* have been detected by fluorescence microscopy of live and fixed cells after incubation with fluorophore-coupled dextran [27]. To further investigate these endocytic compartments, we incubated *S. vortens* and *S. salmonicida* trophozoites with a 10-kDa Dextran-TexasRed fluid phase marker (Fig. 4). In stark contrast to the distinctly arrayed PV/PEC labelling seen in *Giardia lamblia* (Fig. 4A), confocal microscopy revealed the presence of several dispersed labelled organelles in both *S. vortens* (Fig. 4B) and *S. salmonicida* (Fig. 4C). *Spironucleus* spp. display several relatively large globular membrane compartments, similar to those observed in well-characterized model organisms lacking a fixed subcellular localization [5, 62]. While *S. salmonicida* endocytic compartments localize mostly at the cell periphery (Fig. 4C), *S. vortens* organelles present both peripheral and central localizations (Fig. 4B and Additional file 8: Video S4). We also assessed endosome morphology in *T. foetus* using the same labelled dextran-based approach. Similar to *Spironucleus* species, *T. foetus* presents an antero-posterior axis but no attachment organelle nor dorso-ventral polarization. Similar to *Spironucleus* spp., *T. foetus* accumulated the endocytosed fluid phase maker in several globular endocytic compartments (Fig. 4D) consistent with previous reports on vacuolar structures identified by electron microscopy [63].

Taken together, these data show how, in closely related protozoa lacking dorsal-ventral polarization and a dedicated attachment organelle, endocytic organelles appear to have no specific localization. This lends support to the notion that PV/PEC organelle architecture is intimately associated to the emergence of the VD, both structures as adaptations to the mammalian small intestine niche [27].

**Fig. 5:**
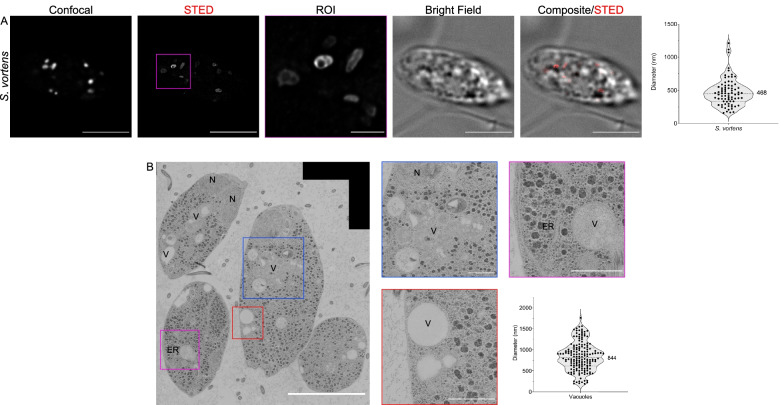
Super-resolution imaging of *S. vortens* endocytic compartments with STED and transmission electron microscopy (TEM). **A** Following incubation with dextran-TexasRed for 30 min, *S. vortens* display elongated endocytic compartments with an average diameter of 468 ± 206 nm with a 95% confidence interval between 421 and 515 nm (upper violin plot, *N* = 10 cells). **B** TEM imaging detects several endosome-like vacuoles (V) throughout the cell, with an average diameter of 844 ± 335 nm with a 95% confidence interval between 763 and 905 nm (lower violin plot). Measurements were done manually. The endoplasmic reticulum (ER) and a nucleus (N) are also highlighted in the images. Scale bars: 5 μm (full field of view) and 1 μm (ROIs)

To visualize and measure the morphological parameters of *Spironucleus* and *T. foetus* endocytic compartments, we performed 2D-STED imaging and transmission electron microscopy (TEM). *S. vortens* cells loaded with 10-kDa Dextran-Alexa Fluor 594 showed accumulation of the fluid phase marker in roughly spherical organelles (Fig. 5A). Labelled endocytic vacuoles have an average diameter of 468 ± 206 nm (95% confidence interval [421; 515] nm, *N* = 10 cells) (Fig. 5A, violin plot). Volumetric rendering of 3D reconstructed optical sections documents the uniformly globular morphology of these organelles (Additional file 9: Video S5). TEM analysis revealed an ellipsoid shape of endocytic organelles in *S. vortens* with an average maximal diameter of 844 ± 335 nm with a 95% confidence interval between [763;905] nm (Fig. 5B, violin-plot). The dimensions measured in TEM represent those of the membrane-delimited organelle. In contrast, the dimensions measured by STED represent a projection of the fluid phase marker distribution within the available organelle lumen. The fact that the former (844 ± 335 nm) is larger than the latter (468 ± 206 nm) indicates that these organelles may contain additional cargo which prevents the endocytosed fluid phase marker to distribute in the complete compartment volume delimited by the organelle membranes. TEM investigation in *S. salmonicida* cells (Additional file 10: Fig. S4A) showed the presence of small globular vacuoles (V) close to the PM (Additional file 10: Fig. S4B) with an average diameter of 205 ± 62.6 nm (*N* = 114) with a 95% confidence interval of 193 and 217 nm (Additional file 10: Fig. S4E). These vacuoles are smaller than the ones found in *S. vortens* (Additional file 10: Fig. S4C, D, F; *p*-value < 0.0001)*.* In these conditions, neither coated vesicles nor a stacked Golgi apparatus could be documented in *S. vortens* or *S. salmonicida*.

**Fig. 6:**
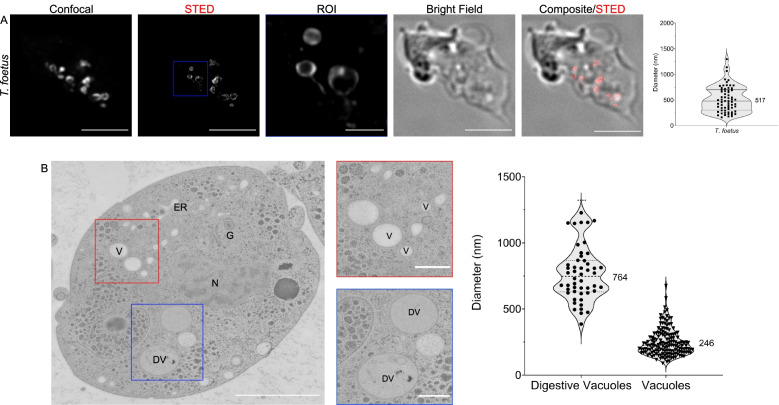
Super-resolution imaging of *T. foetus* endocytic compartments with STED and TEM. **A** 2D-STED analysis of *T. foetus* cells loaded with 10-kDa Dextran-Alexa Fluor 594 illuminates endosomes with globular structures at an average diameter of 517 ± 251 nm with a 95% confidence interval between 455 and 580 nm (upper violin plot, *N* = 10 cells). **B** TEM investigation of *T. foetus* cells reveals two kinds of endosome-like vesicles. Low electron-density vesicles found both at the cell periphery and centre are deemed vacuoles (V) with an average diameter of 246 ± 100 nm (*N* = 153) with a 95% confidence interval of 230 and 262 nm. Vesicles of higher electron density and with noticeable content are digestive vacuoles (DVs). DVs are larger than vacuoles, with an average diameter of 764 ± 203 nm (*N* = 50) with a 95% confidence interval of 707 and 822 nm (lower violin plot). Scale bars: 5 μm (full field of view) and 1 μm (ROIs)

2D-STED analysis of *T. foetus* cells incubated with 10-kDa Dextran-Alexa Fluor 594 revealed a roughly circular distribution of the marker within endocytic vacuoles (Fig. 6A and Additional file 11: Video S6) with an average maximal diameter of 517 ± 251 nm (95% confidence interval [455; 580] nm, *N* = 10 cells) (Fig. 6A, violin plot). TEM imaging revealed the presence of two distinct classes of endosome-like vesicles (Fig. 6B) based on the electron density of the lumen. Low-density vesicles were identified both at the cell periphery and in central areas termed vacuoles (V); vesicles of higher electron density were previously identified as digestive vacuoles (DVs) [63] and contain structured material and membranes. Analysis of TEM micrographs showed that DVs are significantly larger than vacuoles, with an average diameter of 764 ± 203 nm (*N* = 50) (95% confidence interval [707; 822] nm). Vacuoles in turn have an average diameter of 246 ± 100 nm (*N* = 153) in (95% confidence interval [230; 262] nm) (Fig. 6B, violin plot). Stacked Golgi organelles are abundant in TEM micrographs of *T. foetus* trophozoites, as documented previously [64] (Additional file 12: Fig. S5A). Consistent with a more canonical architecture of the membrane trafficking system in *T. foetus*, coated vesicles were observed in the cytosol particularly in the vicinity of Golgi stacks (Additional file 12: Fig. S5B) [63, 65, 66]. These vesicles averaged a diameter of 58.4 ± 13.1 nm (*N* = 128) (95% confidence interval [56.1; 60.7] nm) corresponding to the size range of clathrin-coated vesicles (CCVs) [67]. In our ultrastructure observations, we did not detect multivesicular bodies nor vacuoles containing intra-luminal vesicles, neither in *Spironucleus* spp. nor *T. foetus*.

**Fig. 7:**
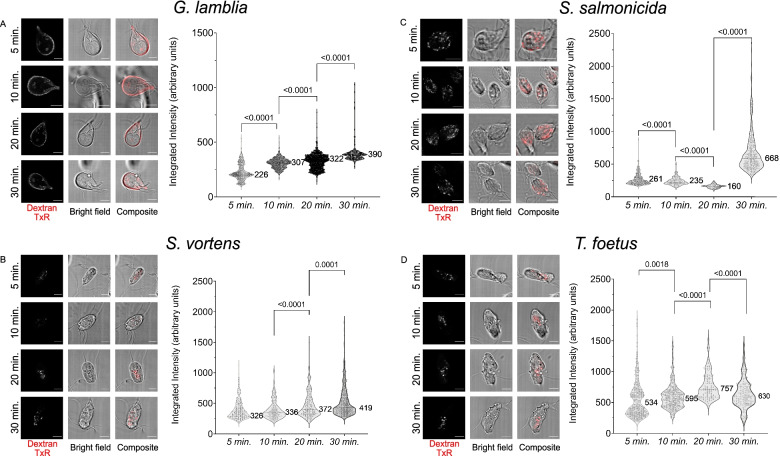
Time course on fluorescent dextran uptake in selected metamonada and discoba specimens: *G. lamblia*, *S. vortens*, *S. salmonicida* and *T. foetus* after 30 min. **A** PV/PECs in *G. lamblia* start acquiring external material right after 5 min of incubation with dextran. Over 30 min of dextran incubation, the endocytic marker does not leave PV/PECs. In **B** *S. vortens*, **C** *S. salmonicida* and **D** *T. foetus*, dextran is up taken in the small vesicles. In *S. vortens*, these small vesicles tend to agglomerate at the centre of the cell while in *S. salmonicida*, these vesicles seem to stay peripheral. In *T. foetus*, vesicles bearing dextran from the periphery of the cell also migrate to the interior of the cell. All scale bars: 5 μm

Finally, to probe the dynamics of endocytic compartments in *G. lamblia*, *S. vortens*, *S. salmonicida* and *T. foetus*, cells were exposed to 10-kDa Dextran-TexasRed for 5, 10, 20 or 30 min, fixed chemically, and imaged by confocal microscopy (Fig. 7). The number of *G. lamblia* PV/PECs labelled with the fluid-phase marker increased over time, with the label accumulating strictly at the cell periphery (Fig. 7A). In contrast, endocytic compartments in *S. vortens* were first visualized at the PM and were then observed at more central locations of the cell at later time points. Given the overall increase in fluorescent intensity and the motile nature of these organelles, it appears there is a constant uptake of dextran over the analysed period (Fig. 7B). In these conditions, *S. salmonicida* and *T. foetus* vacuoles both appear diffused within the cell cytoplasm although *S. salmonicida* organelles show a decrease and then marked increase in dextran content (Fig. 7C) while *T. foetus* organelles present irregular fluctuations in dextran content (Fig. 7D).

### Pan-eukaryotic searches for endocytic markers CHC and CLC reveal the loss of a bona fide CLC within the fornicata lineage and the emergence of putative CLC analogues

**Fig. 8:**
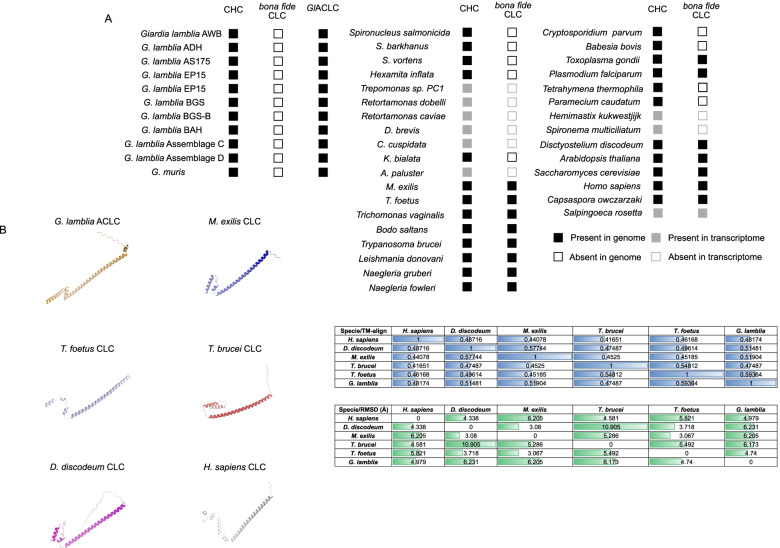
Homology search for bona fide CHC and CLC reveals the key importance of CHC and patchy conservation of CLC. **A** CHC and CLC homology searches demonstrate the conservation of CHC orthologs in all analysed lineages. However, several lineages appeared to have lost a readily detectable bona fide CLC including all selected Fornicata, certain Chromerids [77], some ciliates [78] and members of the Hemimastigophora. **B** Ab initio in silico protein modelling of *G. lamblia* ACLC (formerly *Gl*CLC/Gl4529), *T. brucei* CLC, *T. foetus* CLC, *M. exilis* CLC, *D. discoideum* and *H. sapiens* CLC sequences using AlphaFold, the current standard in in silico protein structure modelling. TM-align and RMSD values were calculated showing a close structure analogy between the predicted structures (table)

Previously, we established that *Giardia* clathrin heavy chain (*Gl*CHC) associates to discrete static foci at the dorsal PM of trophozoites, in close proximity to PV/PECs. Furthermore, *Gl*CHC strongly interacts with a putative albeit highly diverged *Giardia* clathrin light chain homologue previously named *Gl*CLC. Native co-IP experiments demonstrate how CHC is invariably associated to CLC, consistent with the lack of a detectable cytoplasmic pool of CHC and suggesting that virtually all known CHCs and CLCs exist in a complex [26, 27]. However, whether *Gl*CLC is truly a CLC direct homologue (i.e. orthologous) is unclear, as is the prevalence of this protein in other members of the lineage Fornicata. To investigate the occurrence of both CHC and CLC orthologues in selected eukaryotic lineages, we employed protein homology searches based on hidden Markov models (HMM) [68] using as query an alignment of canonical and documented CHC or CLC sequences from several protozoa and metazoan species (Additional file 13: Tables S2 and S5) [69–75]. In this search, we considered assembled read data from RNA-seq experiments (transcriptomics) as reliable as genomic sequence data [76]. In this case, we used the reference CHC or CLC sequences and performed tblastn searches. Nucleotide sequences from each reliable hit (lowest *e*-value) were translated and subjected to a reciprocal blast-p analysis to validate protein identity. We found CHC homologues in all selected genomes and transcriptomes we searched, highlighting the likely essential nature of CHC (Fig. 8A and Additional file 13: Tables S4 and S5).

*Gl*CHC is a clearly divergent ortholog compared to its counterpart in eukaryotic model organisms, with only 24% amino acid identity to human CHC [25]. A domain analysis of selected CHC sequences (Additional file 14: Fig. S6 and Additional file 15: Table S10) reveals that *Gl*CHC contains fewer α-helical domains than other analysed CHC sequences, further highlighting its divergence. We also performed an in-depth search for the CHC triskelion uncoating “QLMLT” motif which we documented previously to be missing in *Giardia* [27, 79, 80]. Notably, this motif appears to be only present in Metazoa and in the closely related Filastera and Choanoflagellata [81–83]. In Fungi, only a partial “L(M)TL” motif was identified, and we were unable to detect a conserved uncoating motif in CHC sequences of members of the Archaeplastida, Amoebozoa or SAR supergroups (Additional file 16: Fig. S7).

**Fig. 9:**
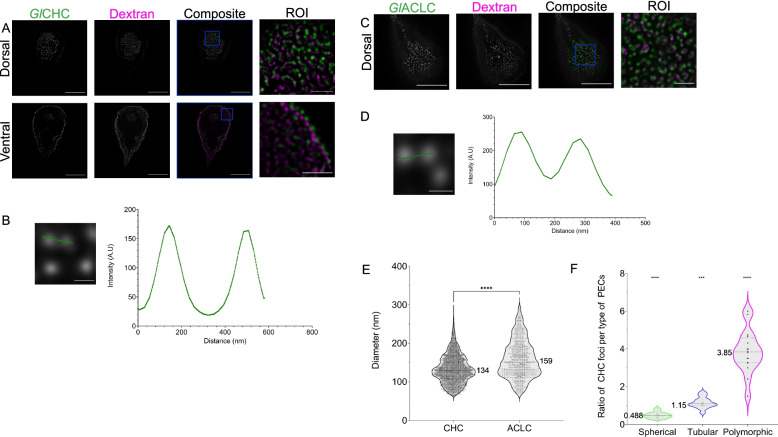
*Gl*CHC and *Gl*ACLC foci associate to *Giardia* PV/PECs with different stoichiometries. **A** An epitope-tagged *Gl*CHC reporter was used to localize the foci of *Gl*CHC deposition at the cell periphery beneath the PM, by STED microscopy. **B** These foci are well-resolved with STED microscopy (left graph). **C** The same was done with an epitope-tagged *Gl*ACLC reporter, and **D** the foci resolved as in **B** (right graph). **E** The foci were segmented with ilastik. Areas were determined and diameters calculated in an automatic procedure assuming spherical geometry. *Gl*CHC foci present an average diameter of 134 ± 36.6 nm (*N* = 4524) with a 95% confidence interval between 132 and 135 nm. *Gl*ACLC foci average a diameter of 159 ± 48.8 nm (*N* = 984) with a 95% confidence interval between 156 and 162 nm. *Gl*ACLC foci are larger than *Gl*CHC in significant statistical manner (*p*-value < 0.0001, *t*-Student test). **F** *Gl*CHC foci associate in different stoichiometry to different classes of PV/PECs. Spherical PV/PECs are either not associated to clathrin or with just one focus: a mean of 0.488 ± 0.159 foci per spherical with a 95% confidence interval between 0.403 and 0.573 foci per spherical organelle. Tubular PV/PECs associate with one focus of *Gl*CHC: a mean of 1.15 ± 0.287 with a 95% confidence interval between 0.994 and 1.3 foci per tubular organelle. Polymorphic PV/PECs associate with 3 or more *Gl*CHC foci with an average of 3.85 ± 1.14 with a 95% confidence interval between 3.25 and 4.46 foci per polymorphic organelle. These distributions are statistically significant with a *p*-value < 0.0001 (ANOVA analysis)

In stark contrast to CHC, the search for bona fide CLC sequences did not retrieve any reliable predictions in available genomes and transcriptomes from species of the Fornicata lineage, including the lineages Hexamitidae, Retortamonas and Carpediemonas-like organisms [59, 75, 84–86]. Importantly, this search did not return the putative, highly diverged *Gl*CLC [27]. There are documented CLC orthologues in members of the Discoba, such as *Trypanosoma brucei* CLC (Tb927.10.14760) [87] and in the parabasalid *Trichomonas vaginalis* (TVAG_29749) [88, 89]. Furthermore, we readily identified a CLC homologue in *T. foetus* (gene accession OHT14195.1, forward HMMer *e*-value of 1.00E−26 and reverse Blastp *e*-value of 2.00E−11, returning the human CLC homologue) (Fig. 9A). Therefore, while bona fide CLC orthologues can be readily identified in Discoba and Euglenozoa members and Preaxostyla—as in the metamonad *Monocercomonoides exilis* [73]—no sequence could be found amongst the members of the Fornicata lineage. Furthermore, we were unable to identify a bona fide CLC sequence within the newly documented transcriptome of Hemimastigophora [90]. Beyond Fornicata, we failed to identify *bona fide* CLC in chromerids such as *Cryptosporidium parvum* and *Babesia bovis* [77] and in some ciliate lineages, such as *Tetrahymena thermophila* and *Paramecium caudatum* [78].

Given that *Gl*CLC’s predicted 3D structure is reminiscent of CLCs (Zumthor et al. [27]) but it could not be retrieved as related to a *bona fide* CLC, with its only known orthologue found in *Giardia muris* (Fig. 8A), *Gl*CLC was further analysed using the HHPred suit, in the attempt to find distantly related non-*Giardia* sequences [91]. This search retrieved no robust prediction for a non-*Giardia* sequence (Additional file 13: Table S9). Given that the degree of divergence is such that no reliable claim to orthology can currently be supported and no orthologue for *Gl*CLC can be found outside the *Giardia* genus, we propose the renaming of *Gl*CLC to *Giardia lamblia* analogous to clathrin light chain—*Gl*ACLC—as a CLC structural analogue acquired and retained in the last *Giardia* common ancestor (LGCA). This appears to correlate with the loss of a bona fide CLC with the last Fornicata common ancestor (LFCA). To test the extent of environmental pressure on this protein family’s evolution, we calculated synonymous vs non-synonymous mutation ratios (*ω* = ks/kn) for *Gl*ACLC homologues (Additional file 17: Fig. S8). Interestingly, known sequences for all *Giardia* isolates present a *ω* < 1 which indicates that current sequences are not under selective pressure to evolve. To further investigate the structural analogy of *Gl*ACLC to canonical CLCs, we performed in silico modelling of its C-terminal domain using the new standard in ab initio protein structure modelling—AlphaFold—based on deep-learning neural networks [92, 93] (Fig. 8B and Additional file 20: Table S11). Template modelling score independent of sequence (Tm-align) and root mean square deviation (RMSD) [94, 95] values provide substantial evidence for the structural analogy of *Gl*ACLC and canonical CLCs, in line with previous observations [27]. The newly predicted structures for *Gl*ACL*C* have a stronger resemblance to the predicted structure of a mammalian clathrin light chain [96]. Altogether, the presented in silico data strongly suggest *Gl*ACLC to be a structural analogue of CLC.

Finally, we asked whether de novo acquisition of a CLC analogue with a divergent sequence, but the preservation of structural features had occurred independently in other Diplomonadida lineages. In a first approach, we selected *S. salmonicida* the closest genetically tractable and sequenced relative to *Giardia* [59, 97] in which a bona fide CLC can not be detected. An epitope-tagged variant of the *ca.* 210 kDa *S. salmonicida* CHC orthologue (*Ss*CHC-3xHA, ORF *Ss*50377_14164) distributes in a punctate pattern throughout the trophozoite cytosol (Additional file 18: Fig. S9A), reminiscent of *Gl*CHC focal assemblies (Additional file 18: Fig. S9B and Additional file 19: Video S7). *Ss*CHC-3xHA was utilized as an affinity handle to define a putative *Ss*CHC interactome in a single native co-IP and protein identification experiment (Additional file 18: Fig. S9C, Additional file 20: Table S11). Amongst several endocytosis-related proteins (*Ss*-dynamin, *Ss*-β-adaptin, *Ss*-calmodulin and *Ss*Sec7), one ORF namely, *Ss*50377_11905, was found to be prominently pulled down and contains several coil-coil domains, at a predicted weight of 39 kDa. The 150-amino acid C-terminus of the protein was modelled in AlphaFold and superimposed with CLC structures (Additional file 18: Fig. S9E). TM-align values within structure similarity (0.5 or above), and RMSD values of *circa* 5-6 Å suggest *Ss*50377_11905 may be a *S. salmonicida* structural CLC analogue (Additional file 21: Fig. S10). Using the *Ss*11905 sequence, we retrieved a putative orthologue only in the available transcriptome of the related diplomonad *Trepomonas* sp. [56, 57], namely, TPC1_16039 (forward tblastn *e*-value of 1E−5 and reverse blastp *e*-value of 4E−12), and in no other selected fornicate lineage (Additional file 22: Table S12).

### GlCHC foci associate with different classes of giardial PV/PECs with varying stoichiometry

Having established the presence of at least three different PV/PEC morphologies, presumably corresponding to different cryptic organelles, and having established that the *Gl*ACLC is a unique *Giardia* protein, we wanted to better understand the association of the clathrin complexes with the various PV/PECs and if there is any correlation between PV/PEC morphology and clathrin assemblies. We first wanted to confirm whether *Gl*CHC and *Gl*ACLC are consistently found together in foci. To address this question, we used STED microscopy to investigate epitope-tagged *Gl*CHC (*Gl*CHC-HA) deposition at distinct foci at the dorsal PM and the cell periphery, consistent with PV/PECs location (Fig. 9A). Segmentation of foci using a ML-assisted ilastik tool allowed to determine the dimensions of *Gl*CHC at an average diameter of 134 ± 36.6 nm (*N* = 4524) (95% confidence interval [132; 135] nm) (Fig. 9B). Similar to *Gl*CHC, the subcellular distribution of epitope-tagged *GlA*CLC-HA showed an identical pattern consistent with its demonstrated direct interaction with *Gl*CHC (Fig. 9C) [27]. Segmentation of foci using a ML-assisted ilastik tool determined the dimensions of *Gl*ACLC foci at an average diameter of 159 ± 48.8 nm (*N* = 984) (95% confidence interval [156; 162] nm) (Fig. 9D). Notably, the average size of *GlA*CLC foci is larger than that of *Gl*CHC foci (*p* < 0.0001, *t*-Student test) (Fig. 9E).

Using STED microscopy, we further determined the number of *Gl*CHC foci showing signal overlap with the three classes of dextran-Texas Red loaded PV/PECs (Fig. 9F). By calculating the degree of the signal overlap between *Gl*CHC foci and PV/PEC lumina, we determined that spherical PV/PECs are associated with at most one *Gl*CHC focus with an average of 0.488 ± 0.159 foci per spherical PV/PEC (95% confidence interval [0.403; 0.573]). Tubular PV/PECs associated with at least one *Gl*CHC focus with an average of 1.15 ± 0.287 foci per tubular PV/PEC (95% confidence interval [0.994; 1.3]). Polymorphic PV/PECs associated with three or more *Gl*CHC foci with an average of 3.85 ± 1.14 foci per PV/PEC (95% confidence interval [3.25; 4.46]). Taken together, we find a directly proportional and statistically significant ratio of clathrin foci to PV/PEC size and type (ANOVA; *p*-value < 0.0001). This is in line with the possibility that PV/PEC morphological heterogeneity is correlated with organelle functional diversity, as measured by association to clathrin assemblies consisting of *Gl*CHC and *Gl*ACLC.

## Discussion

### The *Giardia* endocytic organelle system consists of three classes of membrane compartments

Subsequent to ingestion and excystation, *Giardia* trophozoites attach to the intestinal lumen, proliferating and encysting on localized foci throughout the mucosa of the small intestine [98]. Nutrients required for this propagation are taken up from the environment through PV/PEC-mediated endocytosis of fluid phase and membrane-bound material [24, 27, 30, 99–102]. Despite the essential nature of these endocytic organelles, complete resolution of the ultrastructure of the *Giardia* endocytic pathway remains unsolved. To address this, we performed an ultrastructural investigation of *G. lamblia* endocytic compartments to obtain a nanometric view of their morphology as defined by their membrane as well as the lumen accessible to fluid phase markers in labelling experiments [27, 30].

We began by dissecting an entire *G. lamblia* trophozoite using scanning electron microscopy and focused our analysis on PVs. These structures were segmented and rendered in three dimensions. Using this method unambiguously detected at least two distinct classes of PV morphologies, with some being obviously globular in shape while others presenting a more tubular nature. After expanding our analysis of PVs to super-resolution light microscopy methods STED and STORM [103], we determined that PVs are present in three discernible morphologies: spherical, tubular and polymorphic. Thus, we proposed the renaming of these organelles into peripheral endocytic compartments (PECs).

**Fig. 10:**
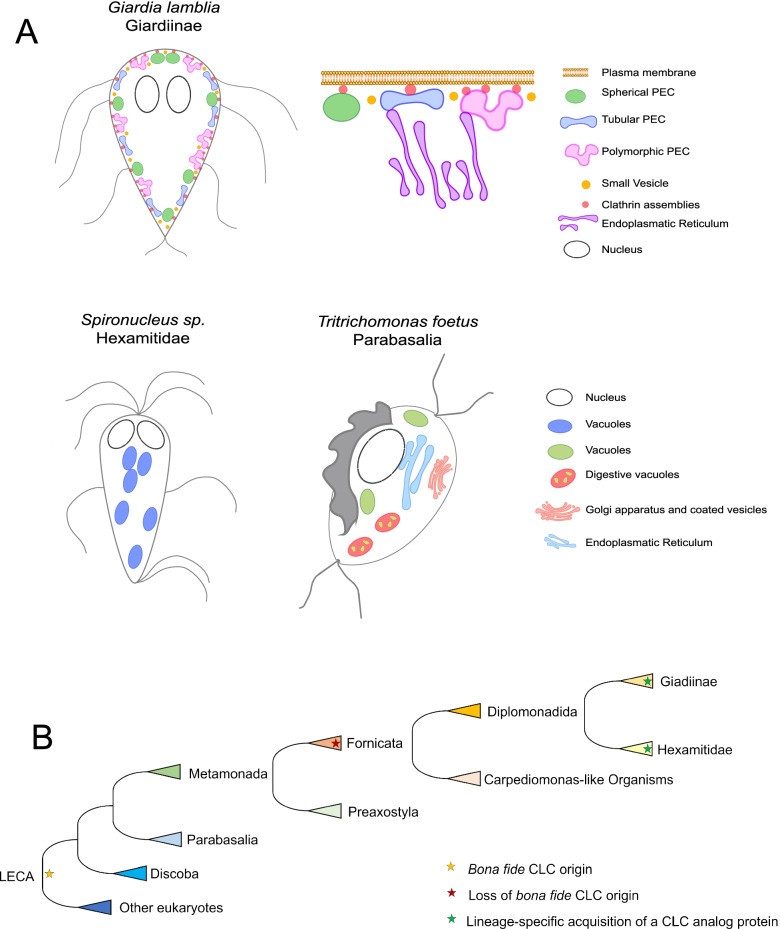
Endosome-like organelle models in *Giardia*, *Spironucleus* and *Tritrichomonas* and proposed evolution of CLC. **A** Simplified cartoons of the endocytic and secretory pathway in *Giardia lamblia* (Giardiinae), *Spironucleus* sp. (Hexamitidae) and *Tritrichomonas foetus* (Parabasalia). PV/PECs are a hallmark of the *Giardia* lineage, while more canonical vesicular endosomes are present in both the *Spironucleus* lineage and Parabasalia. **B** Simplified evolutionary model for bona fide CLC. The last eukaryotic common ancestor possessed a bona fide CLC which was lost at the last Fornicata common ancestor. In at least two derived lineages—Giardinae and *Spironucleus* spp.—de novo protein analogue to CLC was acquired independently

Compared to endosome-like vacuoles in *Carpediemonas*-like organisms (CLOs) [104–106] and large vesicular endosome-like structures observed in *S. salmonicida* and *S. vortens* and the more distantly related Parabasalia member, *T. foetus*, specific and complete remodelling of endosomes has occurred in the *Giardia* genus. *T. foetus*, except for the presence of endosome-like vesicles, presents digestive vacuoles and a stacked Golgi apparatus (Fig. 10). Coated vesicles, likely CCVs, are observed near the *T. foetus* Golgi apparatus and the PM. In our analysis, we could not confirm fluid phase material uptake through the cytostome present in *Spironucleus* sp. [60] and dextran accumulated in spherical vesicles of different dimensions and unknown origin, similar to endosomes. Figure 10A, B summarizes the results of our comparative analysis and highlights the unique endocytic system in *Giardia* where, unlike related species and other excavates, PV/PEC-mediated uptake is restricted to the dorsal side of the cell [27, 107] while the ventral side is deputed to attachment to host structures. Interestingly, endosome and lysosome tubulation has been documented in macrophages [42, 43] and are linked with different physiological states of the organelles and subsequent function in the cell—such as prompting the cell for phagocytosis. This allows for the hypothesis that different kinds of PV/PECs correspond to different stages in organelle maturation/function. In line with this, we provide evidence for different stochiometric associations of CHC foci with different kinds of PV/PECs, although it is possible polymorphic PV/PECs are associated to more foci simply due to their larger area (Fig. 2C).

FIB-SEM ML-based analysis of a full trophozoite also yielded unprecedented views of large numbers of *ca.* 80 nm SVs which had been previously albeit anecdotally reported [27, 108–110]. Based on their electron-dense surface, SVs appear coated and are not related to CHC foci at the PV/PECs-PM interface [26, 27]. Given the absence of a specific marker for these compartments, their identity remains elusive although their coated appearance suggests protein trafficking routes (COPI or COPII) may be involved [25, 111, 112]. However, recent reports in *Giardia* of immuno-EM detection of peroxisome-like proteins in small dense *ca.* 100 nm vesicles [113] provide an open playing field for the definitive identification of these compartments.

### *G. lamblia* possesses a highly divergent clathrin heavy chain and a newly acquired clathrin light chain analogue

We performed an in-depth search for CHC homologues within excavates and other key eukaryotic groups. We found that CHC is conserved in all of these organisms, underlining the vital role of CHC in eukaryotic organisms. The *s*equence divergence of the giardial CHC protein is reflected in an overall decrease in the number of α-helical domains which are essential for the formation of the triskelion leg, and hence necessary for coat assembly [69]. Thus, the reduction in α-helical domains during *Gl*CHC evolution may have led to a lower propensity of *Gl*CHC forming triskelion assemblies and membrane coats. So far, none of the many attempted methods to detect GlCHC in association with small vesicles has been able to show anything other than an exclusive focal localization at PVs/PEC membrane interfaces [27]. Also, the *Gl*CHC protein does not contain the C-terminal uncoating motif “QLMLT” nor is this motif present in the CHC homologues of any diplomonad. In fact, this motif appears to be only present in Metazoa and in the closely related Filastera and Choanoflagellata [81–83] despite the documented ability to form and uncoat bona fide CCVs in some protozoa [114]. In Fungi, only a partial “L(M)TL” motif was identified. We could not detect a conserved uncoating motif in CHC sequences of members of the Archaeplastida, Amoebozoa or SAR supergroups (Additional file 17: Fig. 7). Taken together, this data indicates the uncoating QLMLT motif is apparently specific to and likely and invention of the Holozoa lineage. This observation points to as yet uncharacterized uncoating mechanisms are present in other lineages. For example, clathrin-mediated endocytosis is essential in the parasitic protist *Trypanosoma brucei* and CCVs have been documented in this organism [70, 71, 114, 115]. Clathrin and other coat proteins associated with CCVs need to be recycled. While HSC70 is documented in *T. brucei* and likely involved in clathrin uncoating [80], no bona fide uncoating motif has been documented [71, 87, 114].

In contrast to *Gl*CHC, the evolution of the previously identified putative *Gl*CLC/*Gl*4259 protein presents a different and surprising natural history. This protein was identified as the strongest interactor of GlCHC [27] and is present in all sequenced *Giardia* lineages. *Gl*CLC/*Gl*4259 has no measurable sequence conservation but a high degree of structural similarity to bona fide CLCs, warranting its proposed renaming to *Gl*ACLC. Aside from the *Giardia* genus, we were unable to identify homologues for *Gl*ACLC in any other eukaryotic taxa, nor could we find any orthologues of CLC in any available Fornicata genome/transcriptome sequence, suggesting that the last Fornicata common ancestor (LFCA) lacked a canonical CLC. Taken together, the available data is currently insufficient to decide between two mutually exclusive evolutionary scenarios: (a) secondary loss of a canonical CLC in the last fornicate common ancestor, with acquisition of a structurally and functionally related *Gl*ACLC, or (b) massive sequence divergence of the original, rendering it undetectable by even our most sensitive methods. This latter scenario could have been driven by significant changes in function particularly in Giardia where once-dynamic membrane coating machinery has evolved to become a static structural element supporting interfaces between the plasma membrane and the endocytic system. The discovery of a strong interactor of CHC in the closely related *S. salmonicida Ss*11905, with structural similarity to *GlACLC* as well as to bona fide CLCs is consistent with both scenarios. Notably, this protein neither retrieves *GlACLC* nor CLCs in BLAST searches, leaving no evidence of direct homology. By contrast, robust predictions for CLC homologues were made for members of the Preaxostyla, Discoba and Parabasalia lineages. Although scenario 2 is possible there is no evidence supporting it, and until some emerges, we must favour scenario one, loss and emergence of a convergent structural analogue, which is supported by the evidence in hand. Other lineages appear also to have lost a bona fide CLC, like *C. parvum* and *T. thermophila* [77, 78], but perhaps similar investigations to ours of CHC may identify CLC analogues/divergent homologues. Taken together, this data suggests that the constraints on the CHC primary structure are higher than on CLC even after massive changes in clathrin coat function with demonstrated complete losses in some protists. Members of the *Giardia* genus as well as *S. salmonicida* have no identifiable bona fide CLC, yet at least the giardial *Gl*ACLC has retained its function as a CHC interacting partner.

## Conclusions

Our data provide a robust understanding of *Giardia*, *Hexamitidae* members and *Tritrichomonas foetus* endocytic pathway organellar ultrastructure. Contrary to *Spironucleus* or *Tritrichomonas* and other excavates, *Giardia* underwent a complete remodelling of its endocytic machinery. Our investigation revealed its organelles to be polymorphic in nature, justifying the proposed name change to peripheral endocytic compartments. Furthermore, the analysis of *Gl*CHC sequences highlights its divergence which is likely due to a massive reorganization of the endocytic pathway in these species, while the origin and evolution of CLC structural and to some extent functional homologs in *Giardia* (*GlACLC*) and in certain *Hexamitidae* members (*S. salmonicida* and *Trepomonas* sp. PC1) remains uncertain.

## Methods

### Cell culture and transfection

*Giardia intestinalis* strain WB (clone C6; ATCC catalogue number 50803) trophozoites were grown using standard methods as described in Morf et. al. [116]. Episomally transfected parasites were obtained via electroporation of the circular pPacV-Integ-based plasmid prepared in *E. coli* as described in Zumthor et al. [27] Transfectants were selected using Puromycin (final conc. 50 μg ml^−1^; InvivoGen). *S. vortens* and *S. salmonicida* were cultured as described before [58, 59]. *S. salmonicida* was transfected using a modified PAC vector and selected with Puromycin (final conc. 50 μg ml^−1^; InvivoGen) [97]. *T. foetus* was axenically grown also as described [63].

### Construction of expression vectors

*S. salmonicida* CHC sequence (SS50377_14164) was amplified with the primers ATATTTAATTAAGGCGGATCTATAGTTTCTTGGAATACTAAAATAGGA (forward) and TATGCGGCCGCCACCAGTTATCAGCGGGTGCC (reverse) containing a MluI and a NotI rectrictyion site, respectively. The genomic sequence amplified contained a 5′ UTR region of 179 bp which encodes a putative promoter. The genomic fragment was inserted in the previously described vector pSpiro-PAC-3xHA-C [97].

### Focused ion beam scanning electron microscopy (FIB-SEM) of a full *Giardia* trophozoite and image analysis

Wild-type *Giardia lamblia* trophozoites were subject to high-pressure freezing and processed as established in [27]. Ion milling and imaging were performed in a Auriga 40 Crossbeam system (Zeiss, Oberkochen, Germany) using the FIBICS Nanopatterning engine (Fibics Inc., Ottawa, Canada) following the aforementioned established protocol. The pixel size was set to 5 nm, obtaining isotropic imaging. Alignment of the dataset was performed resorting to the ImageJ plugin Sift [53]. Image segmentation was done using the semi-autonomous algorithm ilastik [38]. The routine of pixel and object classification are used. Algorithm training was performed in a small representative region of the dataset which was then applied to the complete dataset. Imaris (Bitplane AG) was used for three-dimensional rendering and volume measuring. Diameters (*d*) of spherical PV/PECs were measured based on radius (*r*) calculation after volume (*V*_spherical_) determination in Imaris. Thus, ${V}_{\mathrm{spherical}}=\frac{4}{3}\pi {r}^3\leftrightarrow r=\sqrt[3]{\frac{3{V}_{\mathrm{spherical}}}{4\pi }}\leftrightarrow d=2r$. Diameter (*d*) and length (*L*) of tubular PVs/PECs (and assuming a cylindric shape) were calculated after lateral area (*A*) and volume (*V*) determination in Imaris. Thus, *A* = 2*πrL* + 2*πr*^2^. As *L* ≫ *r*, it can be simplified to *A* = 2*πrL*. With $V=\pi {r}^2L\leftrightarrow r=\frac{2V}{A}$ and $L=\frac{A}{2\pi r}$. Finally, *d* = 2*r*. Graphical representations were shown in their log_10_ for easier comparison.

### Transmission electron microscopy analysis of *Giardia lamblia*, *Spironucleus* spp. and *Tritrichomonas foetus* cells and analysis

*G. lamblia*, *S. vortens*, *S. salmonicida* and *Tritrichomonas foetus* samples were subject to high-pressure freezing and processed as we previously established [27, 117]. Samples were imaged in a FEI CM100 transmission electron microscope. The pixel size was assigned to 0.8 nm. Tiles were obtained automatically after the determination of the focal point. Tiles were aligned with TrakEM2 [36].

### Immunofluorescence assays

Chemically fixed cells for subcellular recombinant protein localization were prepared as previously described [118]. HA-epitope tagged recombinant proteins were detected using a rat-derived monoclonal anti-HA antibody (dilution 1:200, Roche) followed by a secondary anti-rat antibody coupled to AlexaFluor 488 fluorophores (dilution 1:200, Invitrogen). Samples were embedded in Vectashield (VectorLabs) or Prolong Diamond Mounting medium (Invitrogen) containing 4′,6-diamidino-2-phenylindole (DAPI) for nuclear staining.

### Fluid-phase marker uptake

Dextran uptake assays were performed as described in [27, 117] using dextran 10 kDa at 2 mg/mL (Invitrogen). Coupled fluorophore was chosen based on the image technique chosen. Immunostaining was performed as described above with the exception of using only 0.02% Triton-X100 (Sigma) in 2% BSA (Sigma) for permeabilization, to prevent leakage and loss of dextran signal. Intensities were calculated with a costume-developed macro in Fiji/ImageJ [53], resorting to WEKA algorithms for segmentation [119].

### Laser scan confocal microscopy (LSCM)

Imaging was performed in an inverted confocal laser scanning microscope Leica SP8 using appropriate parameters. Confocal images were subsequently deconvolved using Huygens Professional (https://svi.nl/Huygens-Professional) and analysed using Fiji/ImageJ [53].

### Stimulated emission depletion (STED) microscopy

Sample preparation was done as described for LSCM. For imaging, samples were mounted in ProLong Diamond antifade reagent (Thermo Fisher Scientific). Super-resolution microscopy was performed on a LSCM SP8 gSTED 3X Leica (Leica Microsystems) using appropriate gating settings. Nuclear labelling was omitted due to possible interference with the STED laser. A pulse depletion laser of 775 nm at 100% strength was used to deplete the signal coming from samples using the fluorophore Alexa Fluor 594. The signal from samples containing Alexa Fluor 488 was depleted with the depletion laser line 592 nm at 50% strength. Pinhole was kept at 1 AU. Images were deconvolved using Huygens Professional (https://svi.nl/Huygens-Professional). After deconvolution, the signal was segmented following a pixel and object classification routine in ilastik. Thresholding was processed in Fiji/ImageJ [53] with the respective calculation of organelle area.

### Single-molecule localization microscopy (SMLM)

Cells were fixed onto a coverslip using a cytospin (6 min, 600 g). Samples were then embedded in Vectashield-based imaging medium [50]. Excess buffer was dried up, and samples were sealed. Single-molecule imaging was performed on a Leica SR-GSD 3D microscope (Leica Microsystems) as described in [120] with cylindrical lenses, in order to image the apical cell region, giving a *z*-depth of about 800 nm. A minimum of 100,000 events were recorded. Image reconstruction was performed with the ImageJ plugin Thunderstorm [52]. Reconstructed images were segmented following a pixel and object classification routine in ilastik [37, 38]. Thresholding and volume calculation was performed in Imaris (Bitplane AG).

### Native co-immunoprecipitation of *S. salmonicida* CHC

Co-immunoprecipitation assays on control wild-type and transgenic *S. salmonicida* bearing the HA-tagged CHC were processed as previously established [27] in non-cross-linking condition agent.

### Protein analysis and sample preparation for mass spectrometry (MS)-based protein identification

SDS-PAGE analysis was performed on 4–10% polyacrylamide gels under reducing conditions. Blotting was done as described in [118] using a primary rat-derived anti-HA antibody (dilution 1:500, Roche) followed by an anti-rat (dilution 1:2000; Southern Biotech) antibody coupled to horseradish peroxidase. Gels for mass spectroscopy (MS) analysis were stained with Instant blue (Expedeon) and de-stained with ultrapure water. MS-based protein identification was performed as previously reported [27].

#### In silico co-immunoprecipitation dataset analysis

The co-IP datasets derived from transgenic cells expressing epitope-tagged “baits” as affinity handles were filtered using dedicated control co-IP datasets generated from non-transgenic wild-type parasites to identify candidate interaction partners unique to bait-specific datasets. This was done using Scaffold4 (http://www.proteomesoftware.com/products/scaffold/). Unless otherwise indicated, bait-derived co-IP data was filtered using high-stringency parameters (exclusive spectrum counts at 95-2-95, 0% FDR) and manually curated to rank putative interaction partners in a semi-quantitative fashion using ESCs as a proxy for relative abundance. Only proteins with more than 10 hits were considered. Proteins in both datasets were only considered if present 3-fold in the transgenic line versus the control. In silico analysis of hypothetical proteins was mainly carried out using BLASTp for protein homology detection (http://blast.ncbi.nlm.nih.gov/Blast.cgi?PAGE=Proteins) and HHPred (http://toolkit.tuebingen.mpg.de/hhpred) for protein homology detection based on hidden Markov model (HMM-HMM) comparisons and a cut-off at *e*-value < 0.05 was implemented to assign in silico annotation to otherwise non-annotated proteins of unknown function [91].

Protein structure was modelled with the ab initio modelling tool AlphaFold (https://alphafold.ebi.ac.uk/) from Alphabet, powered by Google DeepMind (https://deepmind.com/) deep learning neural network algorithms [92, 93]. Modelling was done via Google Colab in a Jupyter notebook environment(//colab.research.google.com/github/deepmind/alphafold/blob/main/notebooks/).

The TM-align calculation was performed online on the server https://zhanggroup.org/TM-score/. Pymol (the PyMOL Molecular Graphics System, version 2.0 Schrödinger, LLC.) was used for protein structure prediction visualization, superimposing and RMSD calculation using the *cealign* command.

### Homologue search and phylogenetic analysis and tree construction

CHC and CLC sequences were probed amongst several available genomes and transcriptomes with special focus within the fornicata members. Query protein sequences for CHC and CLC from several pan-eukaryotic representatives were obtained and aligned using MUSCLE v.3.8.31 [121] (Additional file 13: Table S2). Resulting alignments were used to generate hidden Markov models using the hmmbuild option and HHMer searches were made on all available genomes with an *e*-value cut-off of 0.01 [68]. Hits were considered valid if reciprocal BLASTp returned a *Homo sapiens* homologue with a *e*-value < 0.05. Transcriptome searches were carried out resorting to tBLASTn searches using the *Homo sapiens* and *Monocercomonoides exilis* respective sequences for CHC or CLC. Once a hit was found, it was translated into an amino acid sequence and was considered valid if it pulled a *Homo sapiens* homologue with an *e*-value < 0.05. All found sequences can be found in Additional file 13: Tables S2 to S9. Protein domain searches were performed at the Conservate Domain Database (CDD), through the Pfam database [122, 123]. The interPro and SMART platforms were also used for domain classification [124, 125]. Synonymous vs non-synonymous mutation ratio was calculated with an available online software (http://services.cbu.uib.no/tools/kaks) following maximum likelihood parameters.

### Statistical analysis and further used software

All data was analysed for statistical significance and plotted using the Prism 9 (Graphpad, https://www.graphpad.com/scientific-software/prism/) software. Images were composed using the Affinity Designer software (https://affinity.serif.com/en-gb/). Video processing was made using Da Vinci Resolve v17.3.

## Supplementary Information

**Additional file 1: Fig. S1.** Rendering of a *G. lamblia* trophozoite scanned with FIB-SEM reveals the cell’s inner ultrastructure. (A) 3D view of acquired FIB-SEM trophozoite data. (B) Single slice showing inner cellular structures such as cytoskeleton elements at the median body (MB), the ventral disk (VD), the endoplasmic reticulum (ER), mitosomes (m) and peripheral vacuoles (PV), highlighted in the region of interest (ROI). (C) Segmentation of different categories of the dataset: cell volume (138 μm^3^), cytoskeleton, endoplasmic reticulum, peripheral vacuoles, small vesicles and mitosomes. (D) Mitosome volume (N = 14, violin-plot) was determined post segmentation at an average volume of 0.001093±0.0005698μm^3^ in a 95% confidence interval between [0.0007643, 0.001422] μm^3^.

**Additional file 2: Video S1.** Three-dimensional rendering of endocytic compartments in *G. lamblia* derived from FIB-SEM sectioning and imaging. Scale bar 1 μm.

**Additional file 3: Fig. S2.** Cryo-SEM of freeze-fractured trophozoites reveals varying vacuolar morphology in *Giardia lamblia*. (A) Overview of cryo-preserved *Giardia* trophozoites subjected to freeze-fracture and SEM imaging. Nuclei (N), Endoplasmic Reticulum (ER), Ventral Disk (VD) and peripheral endocytic compartments (PEC) and plasma membrane (PM) are clearly identifiable. (B and C) Insets showing different PV/PEC morphology: vesicular (asterisk) and tubular (hashtag). Scale bar: (A) 2 μm and (B and C) 500 nm.

**Additional file 4: Fig. S3.** TEM investigation of *Giardia lamblia* endocytic and secretory pathway. (A) Overview of a trophozoite. Different PV/PEC structures, vesicular and tubular are observed, together with small vesicles (SV). The N (nucleus) and ER are also highlighted. (B) Close up on tubular PV/PECs (hashtag). (C) Close up on vesicular PV/PECs (asterisk) and SVs (arrowhead). Scale bars: (A) 2 μm, (B) 1 μm and (C) 500 nm.

**Additional file 5: Video S2.** Comparison between confocal and STED imaging of *Giardia* PV/PECs. Scale bar: 3 μm.

**Additional file 6: Video S3.** Tri-dimensional reconstruction of PV/PECs from STORM data.

**Additional file 7: Table S1.** PV/PECs volume comparison as calculated in FIB-SEM and STORM experiments.

**Additional file 8: Video S4.** Tri-dimensional confocal imaging of *S. vortens* with Dextran-Texas Red. Both peripheral and near-nuclear endosome-like vacuoles are observed.

**Additional file 9: Video S5.** Tri-dimensional STED imaging of *S. vortens* with Dextran Alexa Fluor 594 reveals endosome-like vacuoles in greater detail.

**Additional file 10: Fig. S4.** TEM investigation of *S. salmonicida* endocytic and secretory pathway. (A) *S. salmonicida* presents vacuolar formations close to the plasma membrane. Cells also present a prominent endoplasmic reticulum (ER; blue-framed inset). (B) Highlight of vacuolar formations (V) and ER. (C) Second cell displaying an abundance of PV close to its plasma membrane. (D) Highlight of vacuoles (V) and the prominent ER that connects to the plasma membrane (asterisk). (E) *S. salmonicida* PVs average a diameter of 205±62.6 nm (N=114) in a 95% confidence interval of [193;217]. (F) *S. vortens* peripheral vacuoles are larger than *S. salmonicida* vacuoles in a statistically significant manner (p-value < 0.0001). Diameters were manually determined. Scale bars: (A and C) 2 μm and (B and D) 500 nm.

**Additional file 11: Video S6.** Tri-dimensional STED imaging of *T. foetus* with Dextran Alexa Fluor 594 reveals endosome-like vacuoles in greater detail.

**Additional file 12: Fig. S5.** TEM investigation of *T. foetus* Golgi vesicles. (A) More than one Golgi apparatus (G) can be found per cell. These organelles resemble canonical stacked Golgi releasing small coated vesicles. (B) These vesicles average a diameter of 58.4±13.1 nm (N=128) in a 95% confidence interval of [56.1;60.7] nm. Scale bar: (A) 500 nm.

**Additional file 13: Table S2.** Queries used for CHC HHM profile building. **Table S3.** Results from Pan-Eukaryotic search of CHC homologues in available Proteomes. **Table S5.** Queries used for CLC HHM profile building. **Table S6.** Results for *Gl*CLC search in available Giardia genomes. **Table S7.** Results for *bona fide* CLC present in other genomes/transcriptomes. **Table S8.** Comparison of *Gl*CLC with *bona fide* CLC. **Table S9.** Ten best hits from HHPred.

**Additional file 14: Fig. S6.** Pan-Eukaryotic prediction of clathrin heavy chain protein domains. Pfam analysis of predicted protein domains for several clathrin heavy chain proteins sequences from the following species: *Giardia lamblia, Spironucleus vortens, Spironucleus salmonicida, Trepomonas sp., Hexamita inflata, Dysnectes brevis, Kipferlia bialata, Carpediomonas membranifera, Aduncisulcus paluster, Chilomastix cuspidata, Trypanosoma brucei, Naegleria gruberi, Tritrichomonas foetus, Monocercomonoides exilis, Tetrahymena thermophila, Hemimastix kukwestjiik, Chlamydomonas reinhardtii, Dyctiostilium discoideum, Saccharomyces cerevisiae, Caenorhabditis elegans, Homo sapiens, Salpingoeca rosetta, Capsospora owczarzaki* and *Monosiga brevicollis*. A general decrease in domain complexity is observed in excavates compared with higher eukaryotes. CLOs: Carpediomonas-like organisms. Diplom: Diplomonada.

**Additional file 15: Table S10.** Search for domains from Clathrin heavy chain super family repeats.

**Additional file 16: Fig. S7.** The QLMLT motif is exclusive to Holozoa. Alignment of the C-terminii of CHC sequences from selected Opisthokonta, Archaeplastida, Amoebozoa and SAR species highlights the present of the QLMLT uncoating motif only in Holozoa supergroup. The positioning of the QLMLT is highlighted in blue.

**Additional file 17: Fig. S8.** Calculation of *Giardia* ACLC synonymous vs non- synonymous mutation ratio (ω = ks/kn). (A) Phylogenetic tree resulting of maximum likelihood analysis of the *Giardia* ACLC sequences. Each node is represented by a number. (B) Overall ω < 1 indicating there is no selective pressure on *Giardia* ACLC.

**Additional file 18: Fig. S9.** *Ss*CHC is distributed in the cytosol and interacts with a putative light chain structural analogue. (A) *Ss*CHC was tagged C-terminally with three HA tags and immune-localized to the cellcytosol. Signal was observed in 88% of the analyzed cells (N = 171). (B) High resolution imaging of *Ss*CHC using confocal imaging reveals CHC foci. (C) Single native co-IP analysis of HA-tagged *Ss*CHC reporter (predicted at *ca*. 210 kDa) including distribution of the 171 proteins found in higher abundance with respect to a control co-IP experiment using extracts of non-transgenic *Ss* cells. (D) Qualitative immunoblot analysis of samples from the single native co-IP of HA-tagged *Ss*CHC reporter. I: soluble native co-IP input; P: insoluble cell debris post cell lysis; F: native co-IP flow-through; B: anti-HA beads. WT: non-transgenic *Ss* cells. MW: molecular weight. (E) *Ab initio in silico* protein modelling with AlphaFold of *Ss*11905, *Gl*ACLC, T*b*CLC and *Hs*CLC. TM-align and RMSD scores for predicted structures of *Giardia* ACLC, *Trypanosoma brucei* CLC and Ss11905 with respect to *Homo sapiens* CLC show overall structural conservation with respect to a *bona fide* CLC. Scale bars: (A) 20 μm. (B) 5 μm.

**Additional file 19: Video S7.** Tri-dimensional high resolution confocal imaging and representation of *Ss*CHC-3xHA foci in the cell cytoplasm.

**Additional file 20: Table S11.** *Ss*CHC co-IP results.

**Additional file 21: Fig. S10.** *Trepomonas* sp. PC1 also harbours a putative CLC analogue. *Ab initio* protein modelling of TPC1_16039, orthologous to *Ss*11905 in combination with *Ss*11905, *Gl*ACLC, *Tb*CLC and *Hs*CLC. RMSD and TM-align cores show overall structural conservation with respect to a *bona fide* CLC.

**Additional file 22: Table S12.** Selected CLC-related sequences used for predictive *in silico* modelling analyses.

## Data Availability

All data and materials are available. Access to raw mass spectrometry data is provided through the ProteomeXchange Consortium on the PRIDE platform [126]. Data is freely available using the project accession number and project DOI PXD020201 (https://www.ebi.ac.uk/pride/archive/projects/PXD020201).
