# Supplementary figures and images for: Combined nanometric and phylogenetic analysis of unique endocytic compartments in Giardia lamblia sheds light on the evolution of endocytosis in Metamonada
Source: BMC Biol. 2022 Sep 21;20:206. doi: 10.1186/s12915-022-01402-3 (PMC9490929; doi:10.1186/s12915-022-01402-3)

# Supplemental figure 1

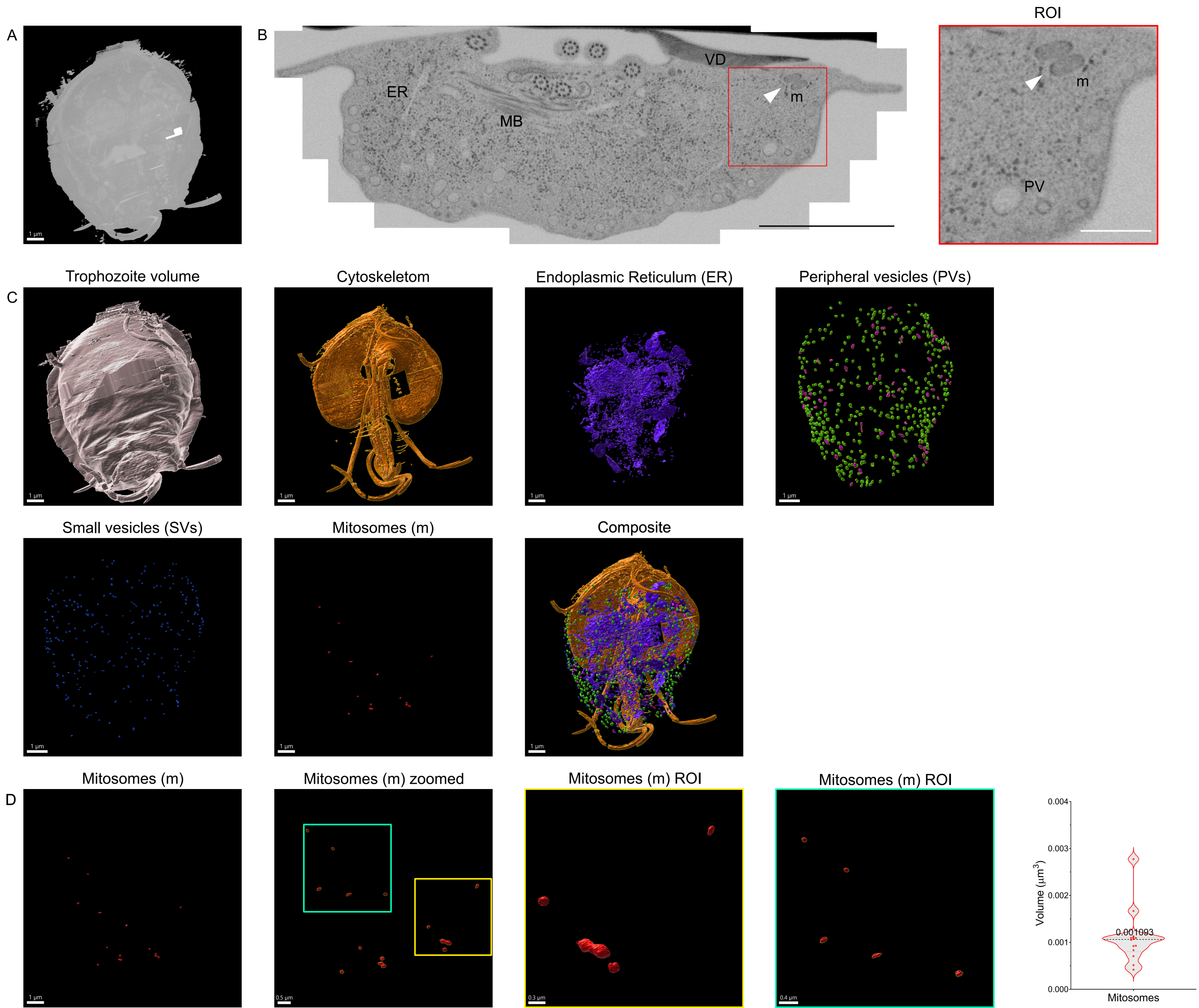

Supplement: Supplementary file 1 — Additional file 1: Fig. S1. Rendering of a G. lamblia trophozoite scanned with FIB-SEM reveals the cell’s inner ultrastructure. (A) 3D view of acquired FIB-SEM trophozoite data. (B) Single slice showing inner cellular structures such as cytoskeleton elements at the median body (MB), the ventral disk (VD), the endoplasmic reticulum (ER), mitosomes (m) and peripheral vacuoles (PV), highlighted in the region of interest (ROI). (C) Segmentation of different categories of the dataset: cell volume (138 μm3), cytoskeleton, endoplasmic reticulum, peripheral vacuoles, small vesicles and mitosomes. (D) Mitosome volume (N = 14, violin-plot) was determined post segmentation at an average volume of 0.001093±0.0005698μm3 in a 95% confidence interval between [0.0007643, 0.001422] μm3. [file 12915_2022_1402_MOESM1_ESM.pdf]

Supplementary Figure 2

A

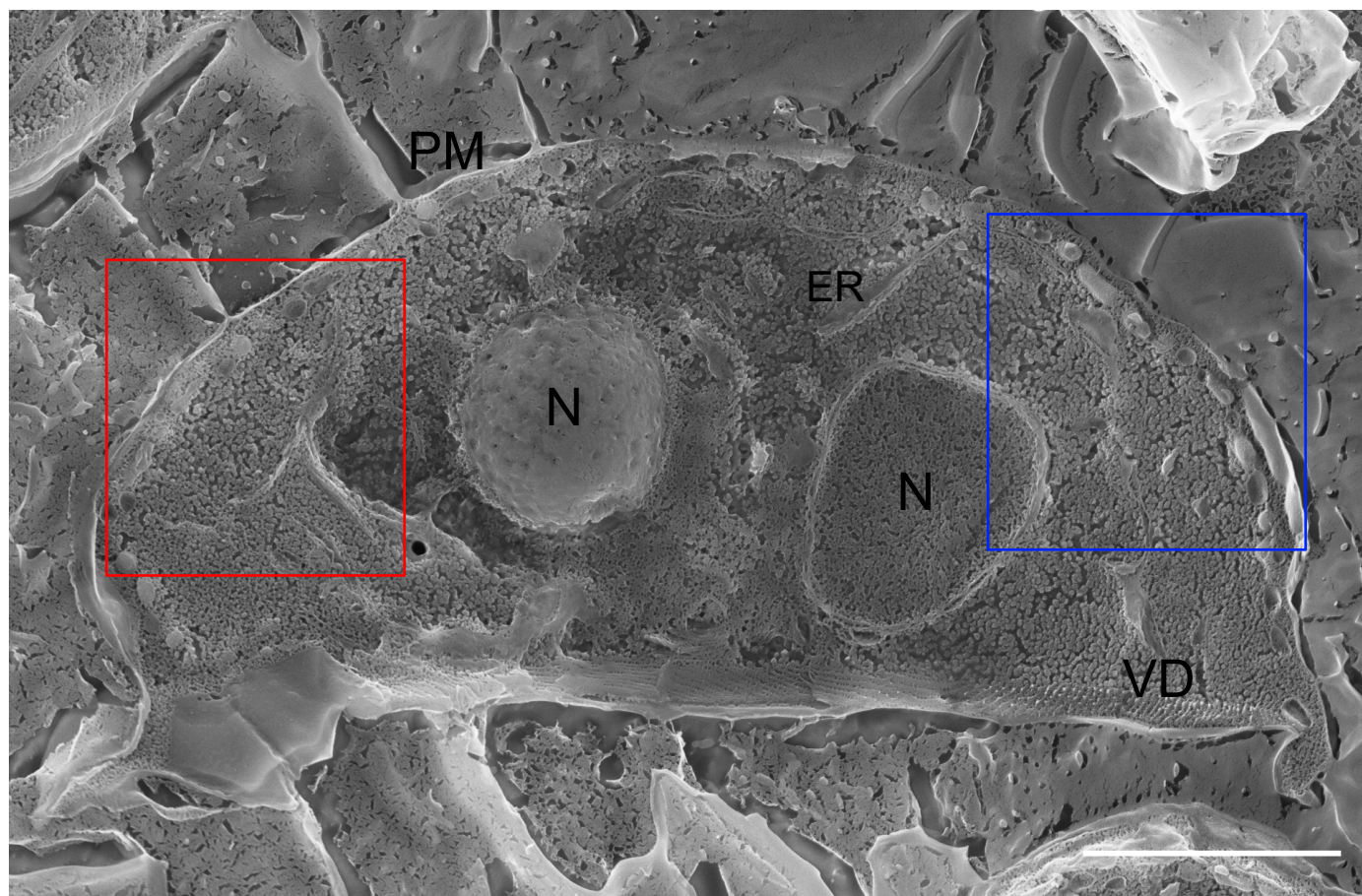

B

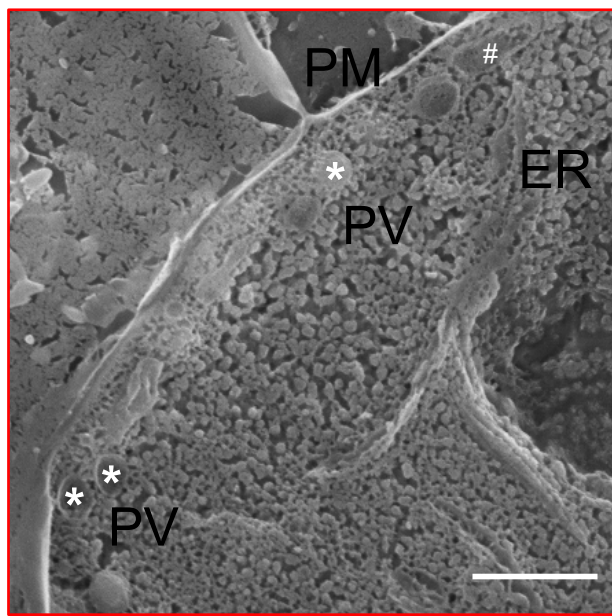

C

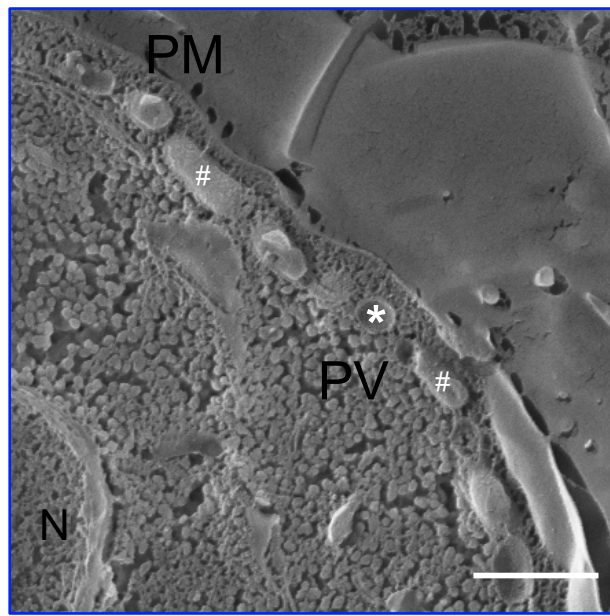

Supplement: Supplementary file 3 — Additional file 3: Fig. S2. Cryo-SEM of freeze-fractured trophozoites reveals varying vacuolar morphology in Giardia lamblia. (A) Overview of cryo-preserved Giardia trophozoites subjected to freeze-fracture and SEM imaging. Nuclei (N), Endoplasmic Reticulum (ER), Ventral Disk (VD) and peripheral endocytic compartments (PEC) and plasma membrane (PM) are clearly identifiable. (B and C) Insets showing different PV/PEC morphology: vesicular (asterisk) and tubular (hashtag). Scale bar: (A) 2 μm and (B and C) 500 nm. [file 12915_2022_1402_MOESM3_ESM.pdf]

Supplementary Figure 3

A

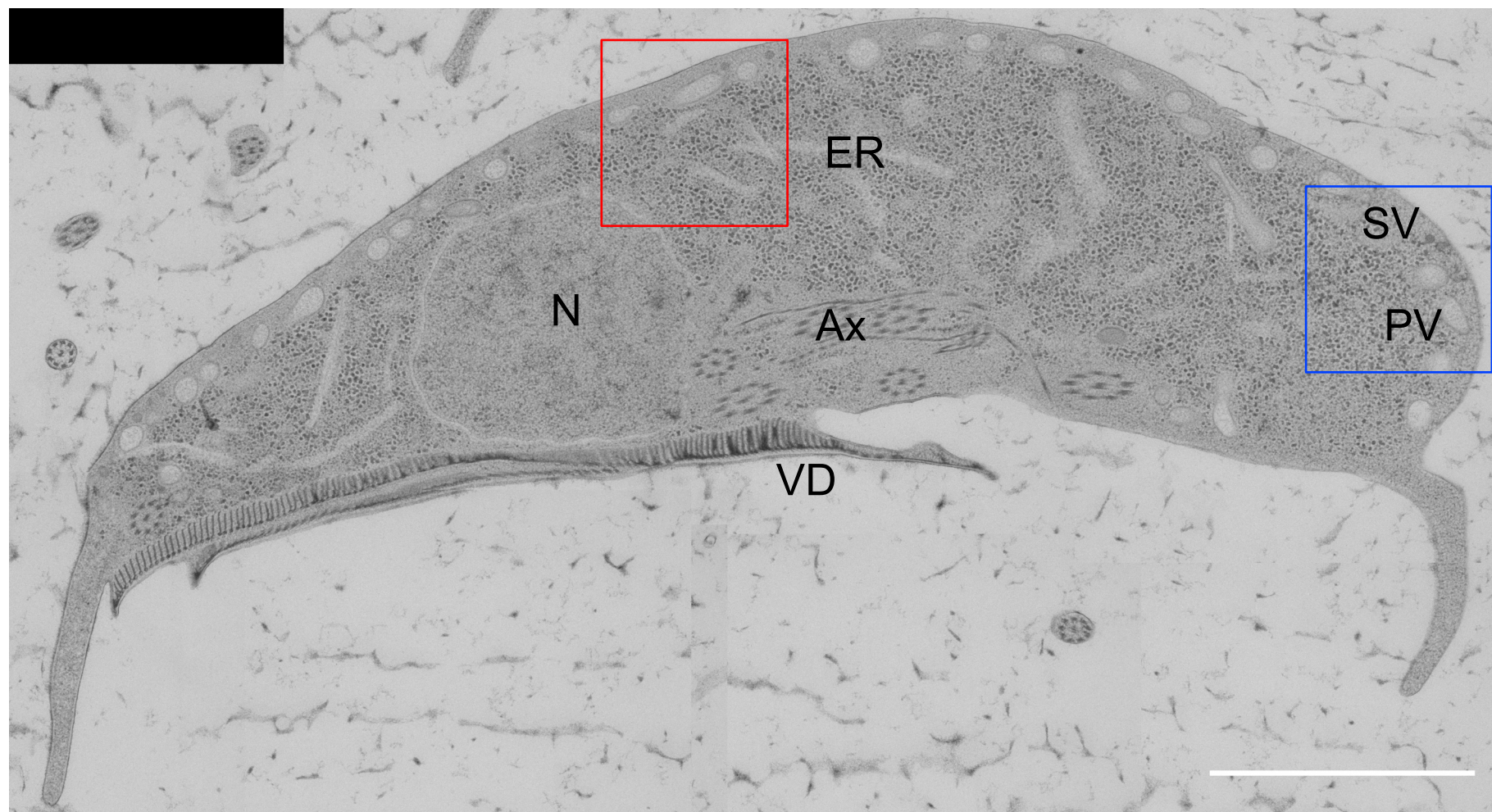

B

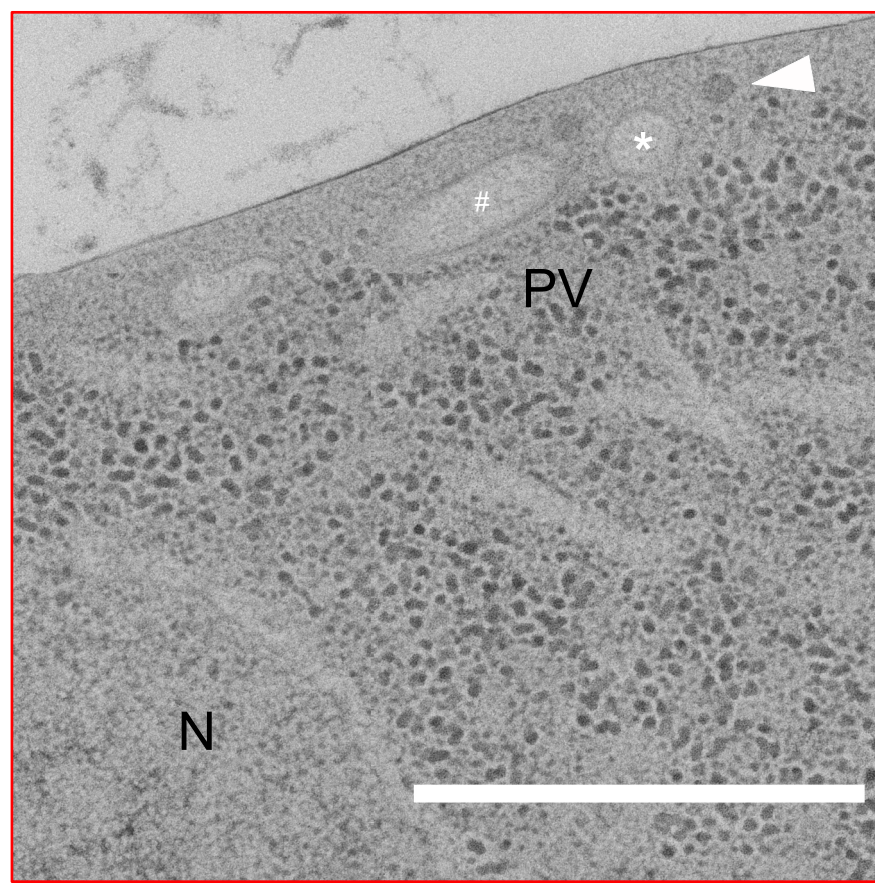

C

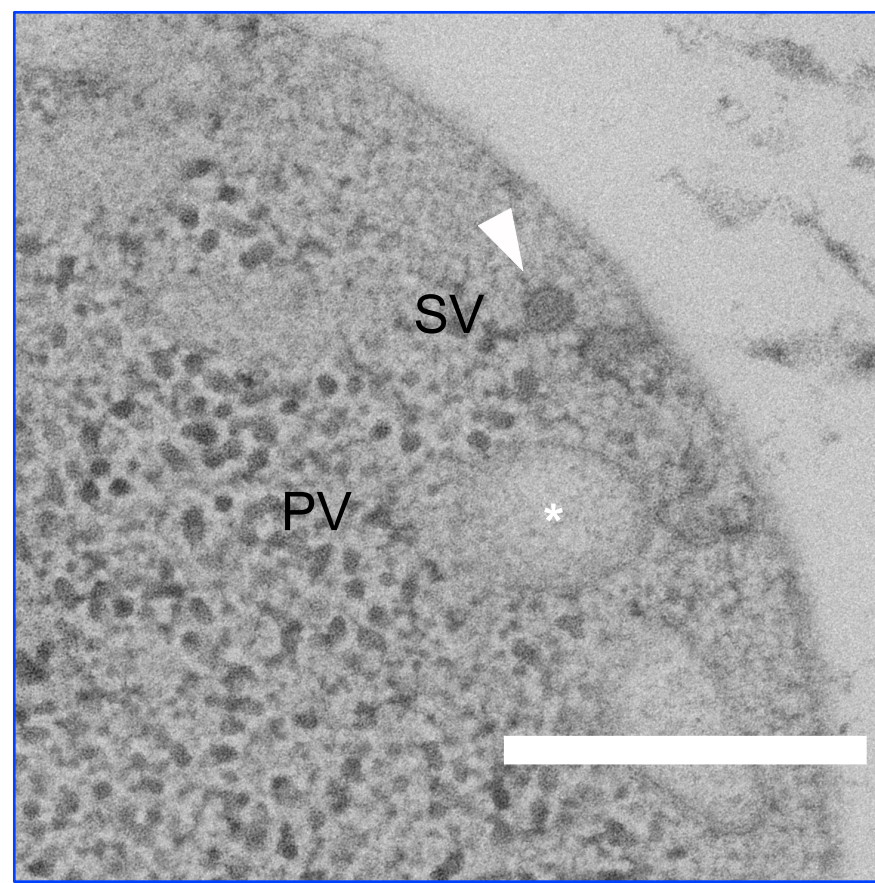

Supplement: Supplementary file 4 — Additional file 4: Fig. S3. TEM investigation of Giardia lamblia endocytic and secretory pathway. (A) Overview of a trophozoite. Different PV/PEC structures, vesicular and tubular are observed, together with small vesicles (SV). The N (nucleus) and ER are also highlighted. (B) Close up on tubular PV/PECs (hashtag). (C) Close up on vesicular PV/PECs (asterisk) and SVs (arrowhead). Scale bars: (A) 2 μm, (B) 1 μm and (C) 500 nm. [file 12915_2022_1402_MOESM4_ESM.pdf]

Supplementary Figure 4

A

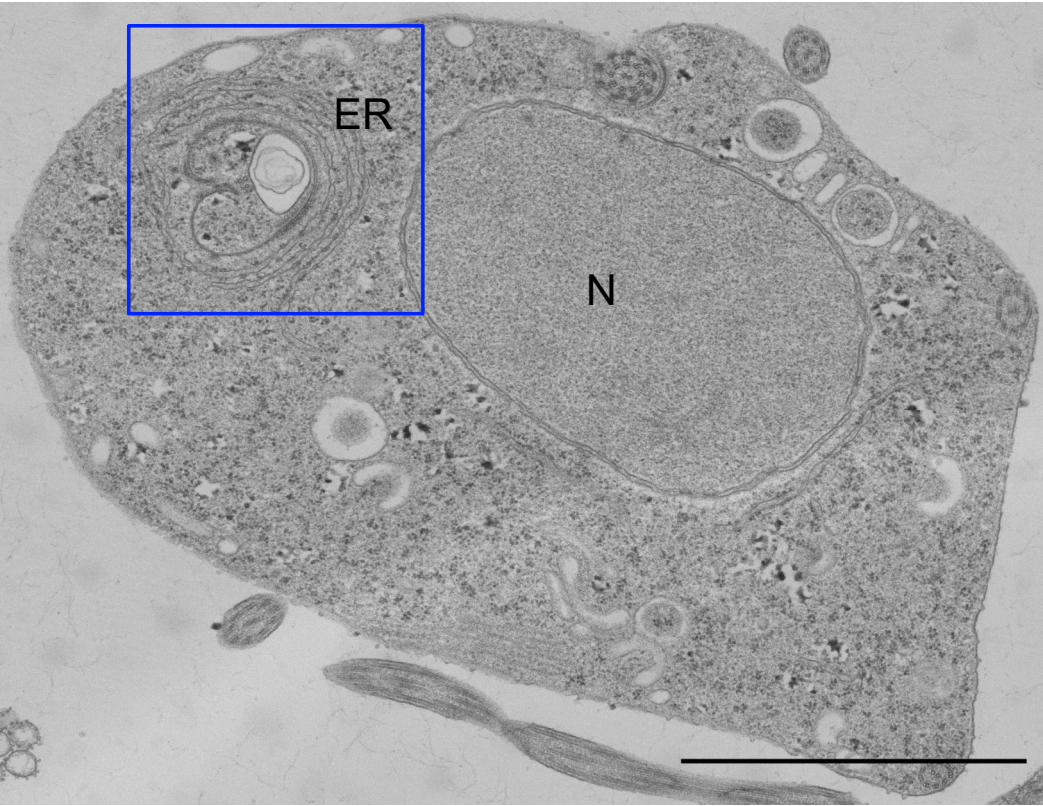

C

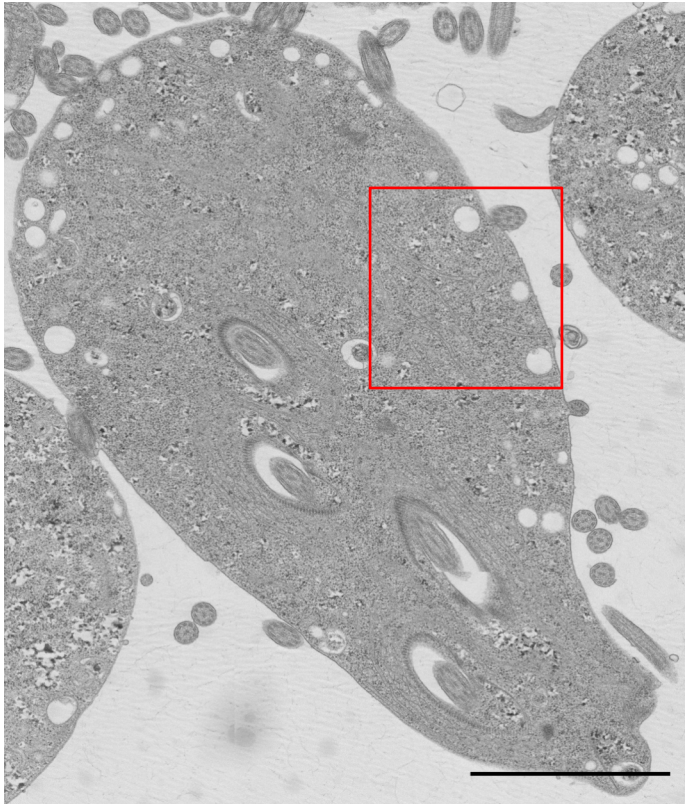

B

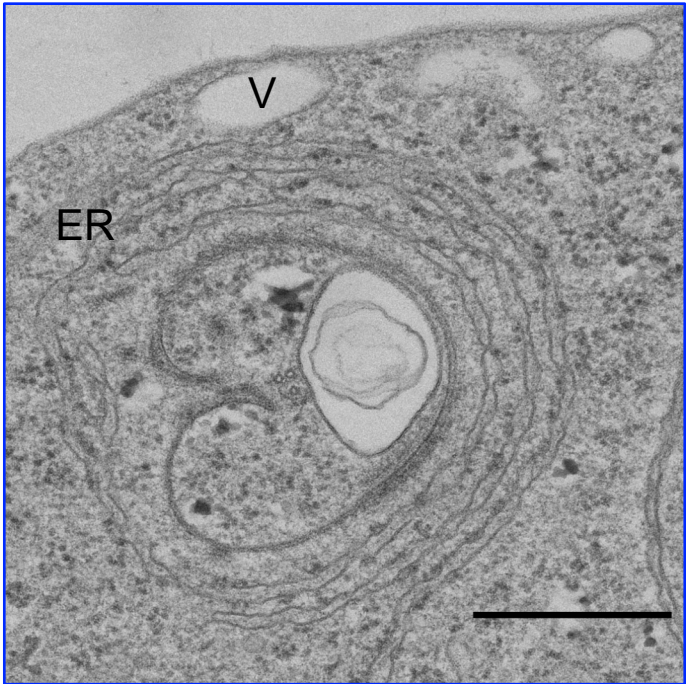

D

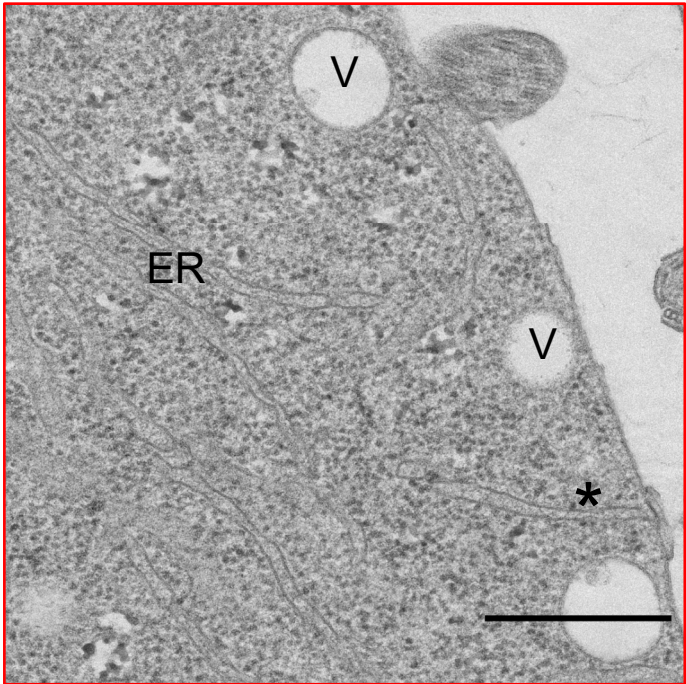

E

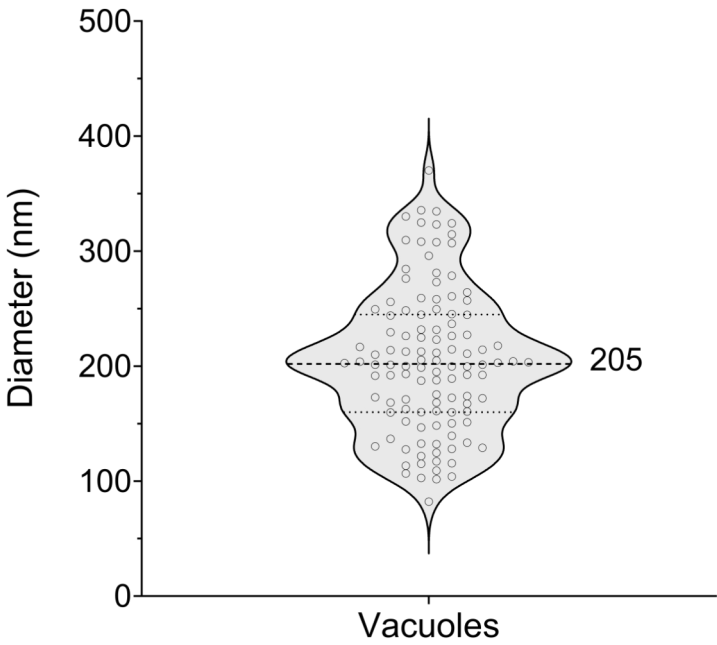

F

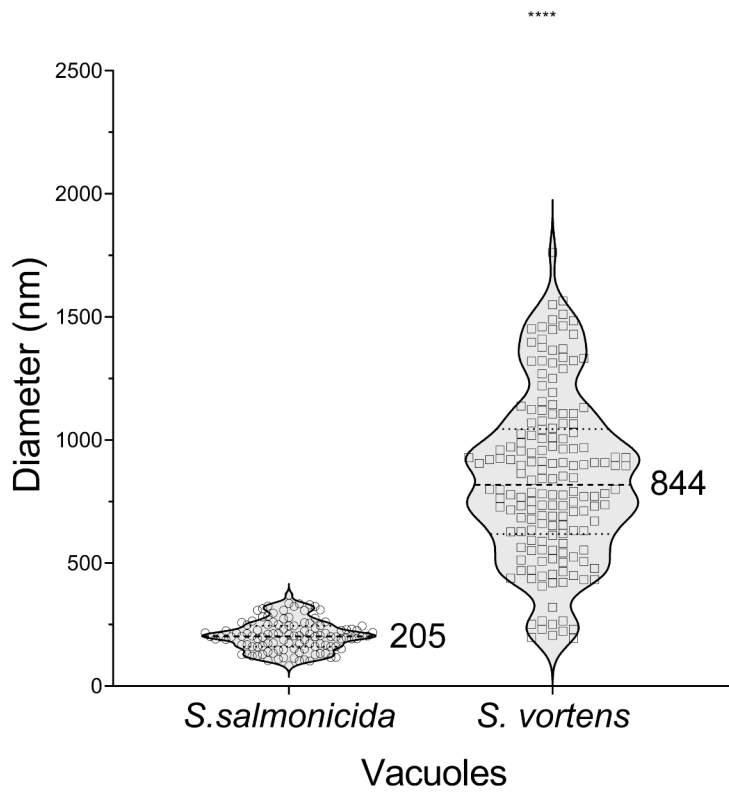

Supplement: Supplementary file 10 — Additional file 10: Fig. S4. TEM investigation of S. salmonicida endocytic and secretory pathway. (A) S. salmonicida presents vacuolar formations close to the plasma membrane. Cells also present a prominent endoplasmic reticulum (ER; blue-framed inset). (B) Highlight of vacuolar formations (V) and ER. (C) Second cell displaying an abundance of PV close to its plasma membrane. (D) Highlight of vacuoles (V) and the prominent ER that connects to the plasma membrane (asterisk). (E) S. salmonicida PVs average a diameter of 205±62.6 nm (N=114) in a 95% confidence interval of [193;217]. (F) S. vortens peripheral vacuoles are larger than S. salmonicida vacuoles in a statistically significant manner (p-value < 0.0001). Diameters were manually determined. Scale bars: (A and C) 2 μm and (B and D) 500 nm. [file 12915_2022_1402_MOESM10_ESM.pdf]

Supplementary Figure 5

A

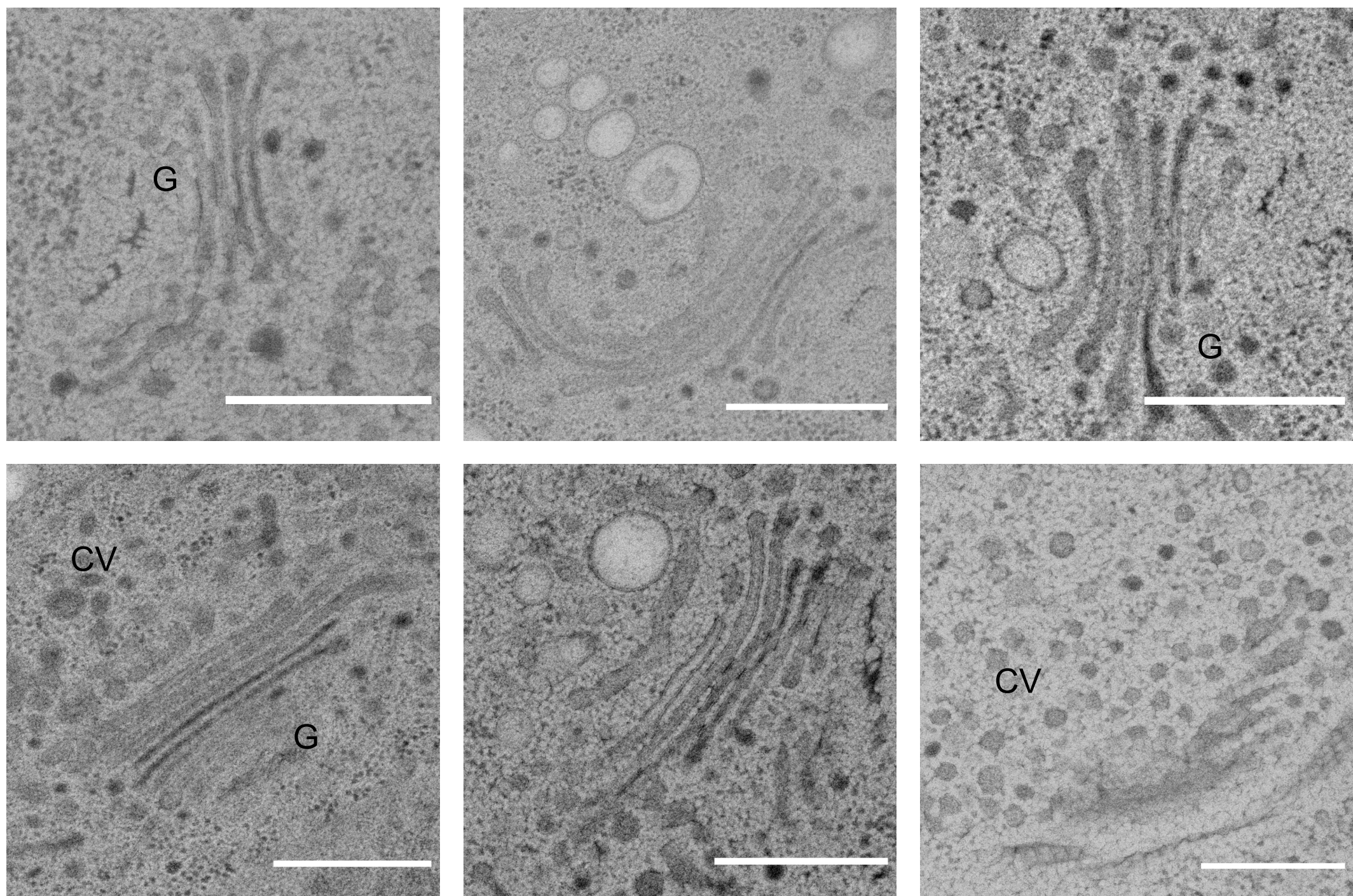

B

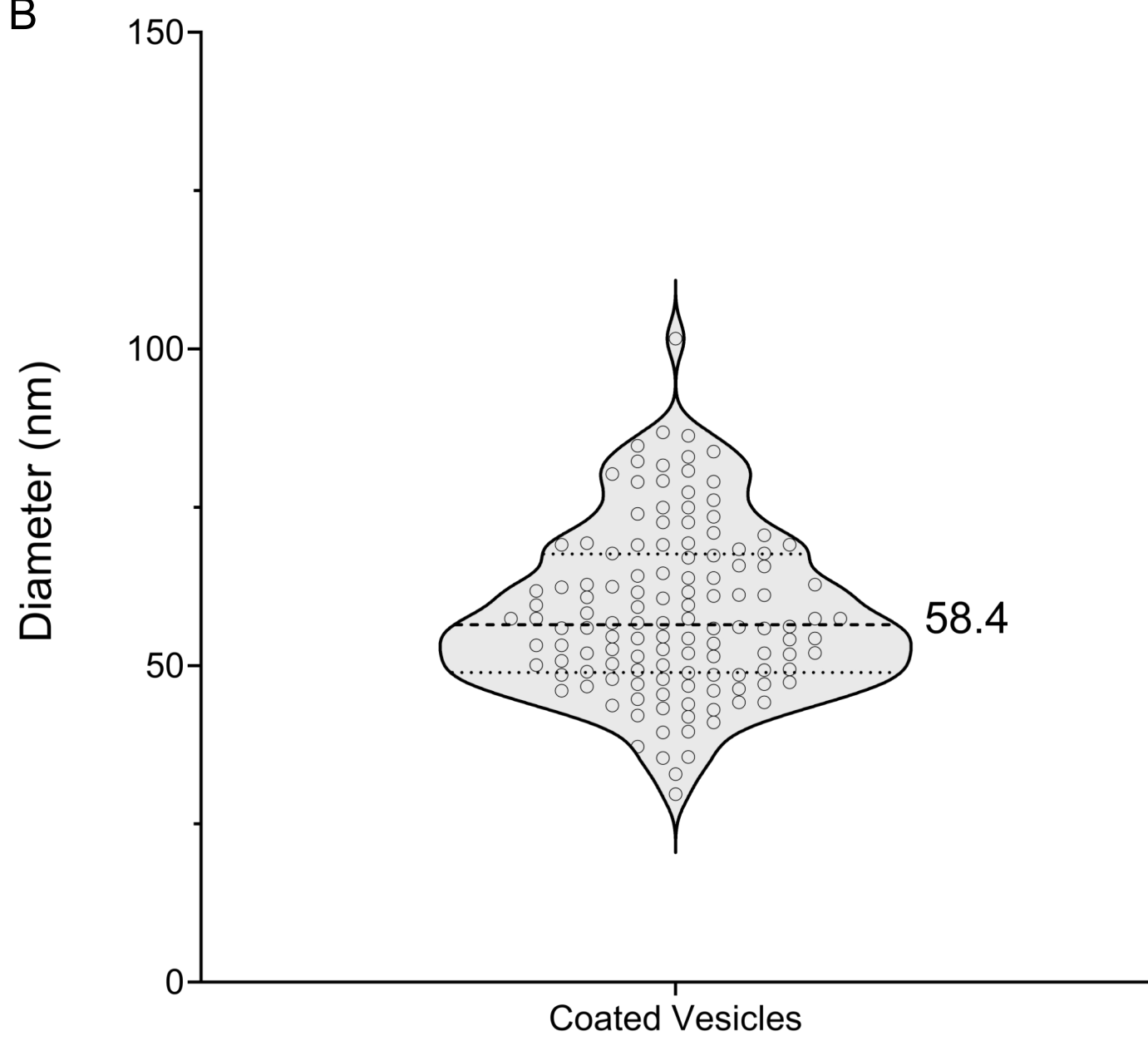

Supplement: Supplementary file 12 — Additional file 12: Fig. S5. TEM investigation of T. foetus Golgi vesicles. (A) More than one Golgi apparatus (G) can be found per cell. These organelles resemble canonical stacked Golgi releasing small coated vesicles. (B) These vesicles average a diameter of 58.4±13.1 nm (N=128) in a 95% confidence interval of [56.1;60.7] nm. Scale bar: (A) 500 nm. [file 12915_2022_1402_MOESM12_ESM.pdf]

Supplementary Figure 6

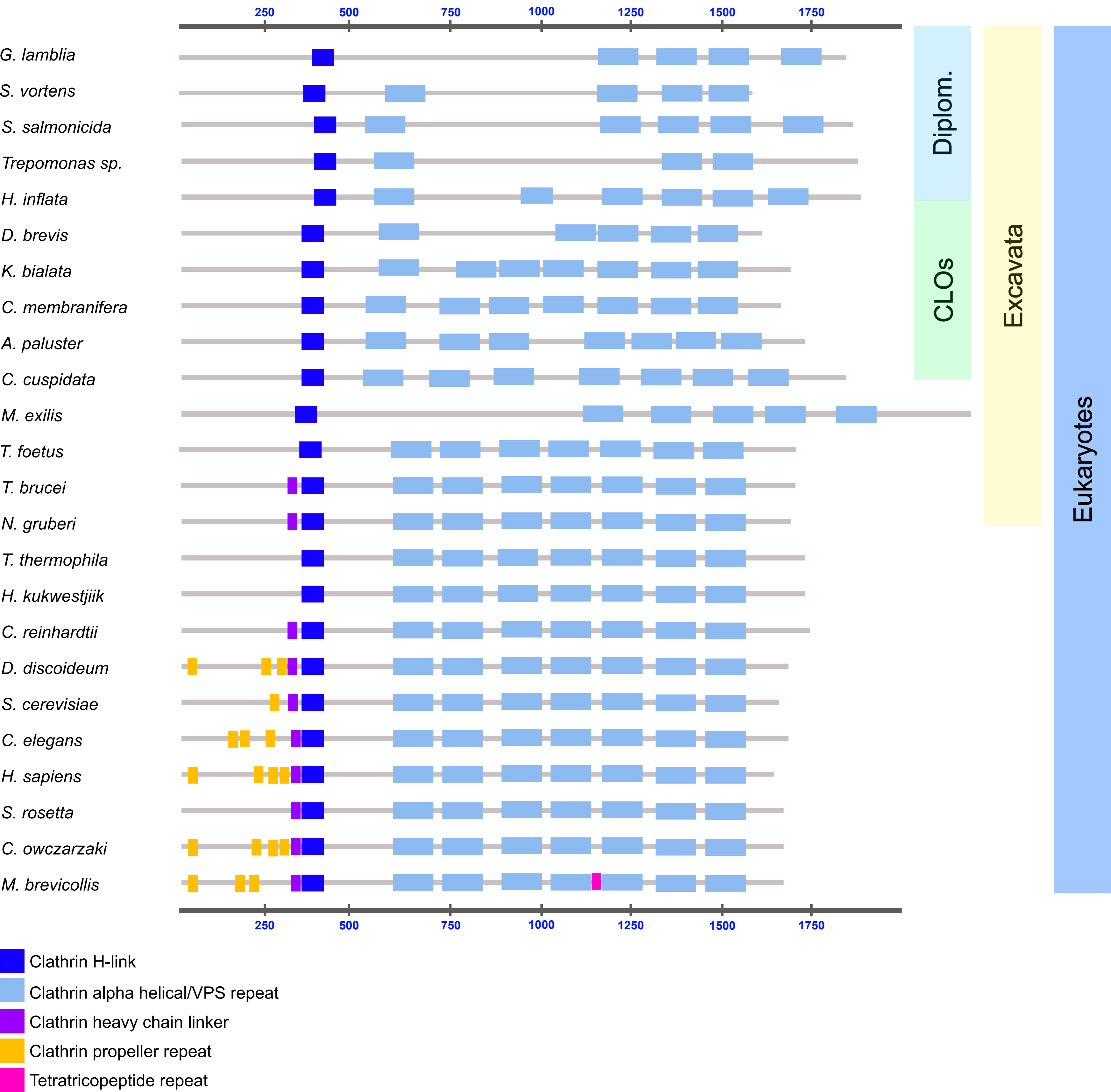

Supplement: Supplementary file 14 — Additional file 14: Fig. S6. Pan-Eukaryotic prediction of clathrin heavy chain protein domains. Pfam analysis of predicted protein domains for several clathrin heavy chain proteins sequences from the following species: Giardia lamblia, Spironucleus vortens, Spironucleus salmonicida, Trepomonas sp., Hexamita inflata, Dysnectes brevis, Kipferlia bialata, Carpediomonas membranifera, Aduncisulcus paluster, Chilomastix cuspidata, Trypanosoma brucei, Naegleria gruberi, Tritrichomonas foetus, Monocercomonoides exilis, Tetrahymena thermophila, Hemimastix kukwestjiik, Chlamydomonas reinhardtii, Dyctiostilium discoideum, Saccharomyces cerevisiae, Caenorhabditis elegans, Homo sapiens, Salpingoeca rosetta, Capsospora owczarzaki and Monosiga brevicollis. A general decrease in domain complexity is observed in excavates compared with higher eukaryotes. CLOs: Carpediomonas-like organisms. Diplom: Diplomonada. [file 12915_2022_1402_MOESM14_ESM.pdf]
