# Supplementary material for: Combined nanometric and phylogenetic analysis of unique endocytic compartments in Giardia lamblia sheds light on the evolution of endocytosis in Metamonada
Source: BMC Biol. 2022 Sep 21;20:206. doi: 10.1186/s12915-022-01402-3 (PMC9490929; doi:10.1186/s12915-022-01402-3)

Supplementary Figure 7

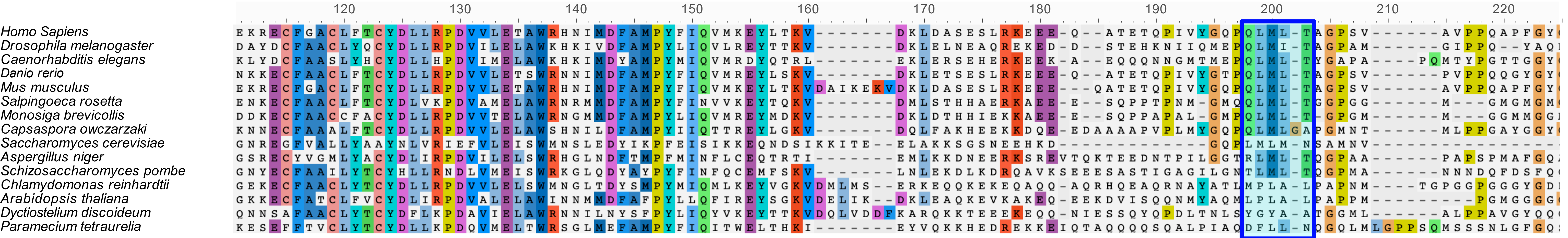

Supplement: Supplementary file 16 — Additional file 16: Fig. S7. The QLMLT motif is exclusive to Holozoa. Alignment of the C-terminii of CHC sequences from selected Opisthokonta, Archaeplastida, Amoebozoa and SAR species highlights the present of the QLMLT uncoating motif only in Holozoa supergroup. The positioning of the QLMLT is highlighted in blue. [file 12915_2022_1402_MOESM16_ESM.pdf]
