# Supplementary material for: Combined nanometric and phylogenetic analysis of unique endocytic compartments in Giardia lamblia sheds light on the evolution of endocytosis in Metamonada
Source: BMC Biol. 2022 Sep 21;20:206. doi: 10.1186/s12915-022-01402-3 (PMC9490929; doi:10.1186/s12915-022-01402-3)
Supplement: Supplementary file 17 — Additional file 17: Fig. S8. Calculation of Giardia ACLC synonymous vs non- synonymous mutation ratio (ω = ks/kn). (A) Phylogenetic tree resulting of maximum likelihood analysis of the Giardia ACLC sequences. Each node is represented by a number. (B) Overall ω < 1 indicating there is no selective pressure on Giardia ACLC. [file 12915_2022_1402_MOESM17_ESM.pdf]

Supplementary Figure 8

A

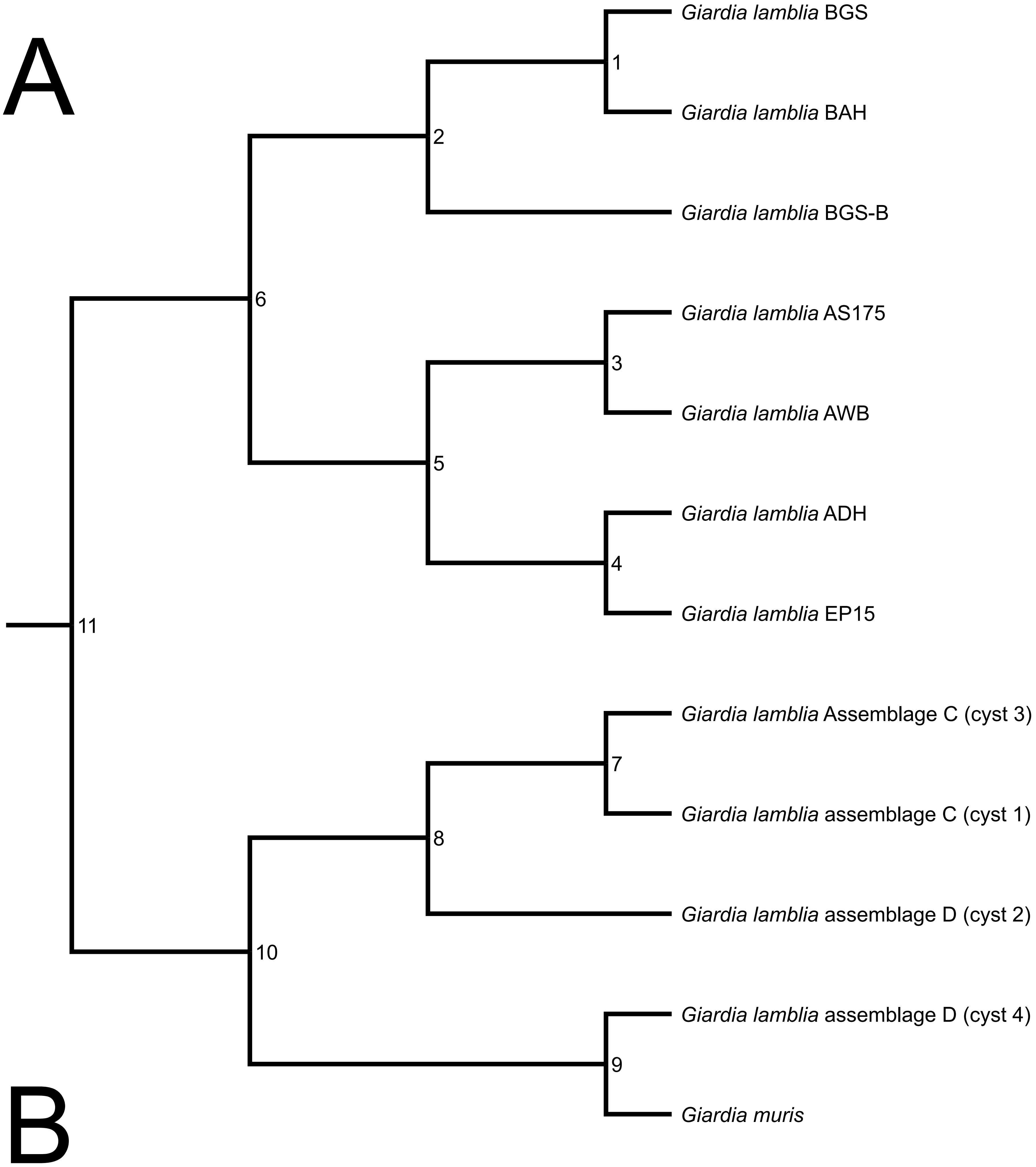

B

Node#	Ka/Ks Branch1	Ka Branch1	Ks Branch1	Ka/Ks Branch2	Ka Branch2	Ks Branch2
1	0	0	1.00E-10	1.2214	0.00389907	0.00319237
2	0	0	1.00E-10	0	0	1.00E-10
3	5.768	0.00576798	1.00E-10	0.6311	0.00851468	0.01349214
4	1.3124	0.02039667	0.01554157	0.7171	0.05336926	0.0744284
5	1.0865	0.01310214	0.01205889	1.0467	0.01439433	0.01375237
6	1.9428	0.06120556	0.03150349	1.2956	0.08488583	0.06551726
7	0	0	1.00E-10	1.2666	0.00126655	1.00E-10
8	0.4003	0.02242913	0.05603452	0.8672	0.06255149	0.07213158
9	0.04253727	0.0205944	0.4841	0.773	0.5227	0.6762
10	0.0583616	0.01207226	0.2069	0.03765873	0.01532114	0.4068
11	0.07482582	0.04253048	0.5684	0.05383007	0.04210393	0.7822
