# Supplementary material for: Combined nanometric and phylogenetic analysis of unique endocytic compartments in Giardia lamblia sheds light on the evolution of endocytosis in Metamonada
Source: BMC Biol. 2022 Sep 21;20:206. doi: 10.1186/s12915-022-01402-3 (PMC9490929; doi:10.1186/s12915-022-01402-3)
Supplement: Supplementary file 18 — Additional file 18: Fig. S9. SsCHC is distributed in the cytosol and interacts with a putative light chain structural analogue. (A) SsCHC was tagged C-terminally with three HA tags and immune-localized to the cellcytosol. Signal was observed in 88% of the analyzed cells (N = 171). (B) High resolution imaging of SsCHC using confocal imaging reveals CHC foci. (C) Single native co-IP analysis of HA-tagged SsCHC reporter (predicted at ca. 210 kDa) including distribution of the 171 proteins found in higher abundance with respect to a control co-IP experiment using extracts of non-transgenic Ss cells. (D) Qualitative immunoblot analysis of samples from the single native co-IP of HA-tagged SsCHC reporter. I: soluble native co-IP input; P: insoluble cell debris post cell lysis; F: native co-IP flow-through; B: anti-HA beads. WT: non-transgenic Ss cells. MW: molecular weight. (E) Ab initio in silico protein modelling with AlphaFold of Ss11905, GlACLC, TbCLC and HsCLC. TM-align and RMSD scores for predicted structures of Giardia ACLC, Trypanosoma brucei CLC and Ss11905 with respect to Homo sapiens CLC show overall structural conservation with respect to a bona fide CLC. Scale bars: (A) 20 μm. (B) 5 μm. [file 12915_2022_1402_MOESM18_ESM.pdf]

Supplemental Figure 9

A

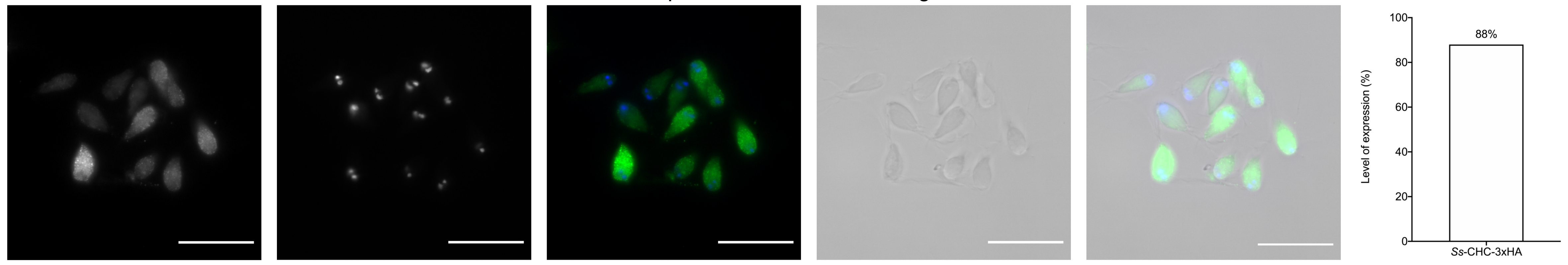

B

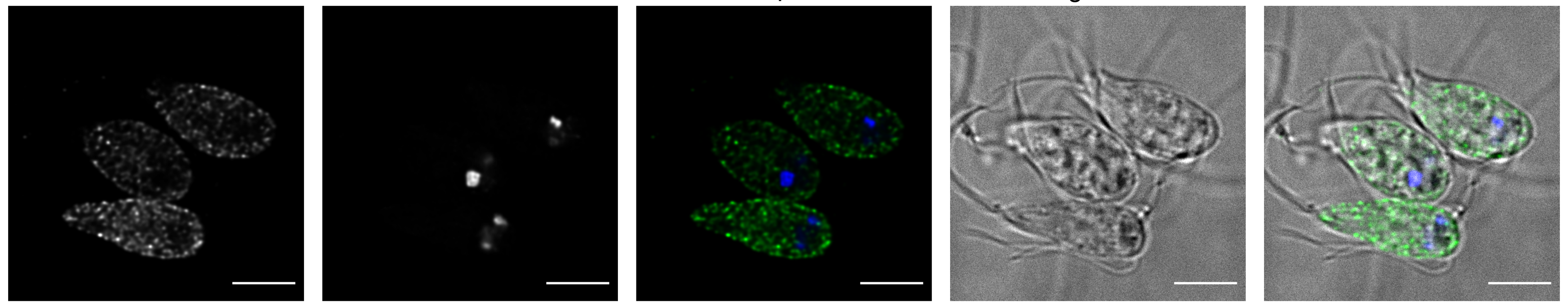

C

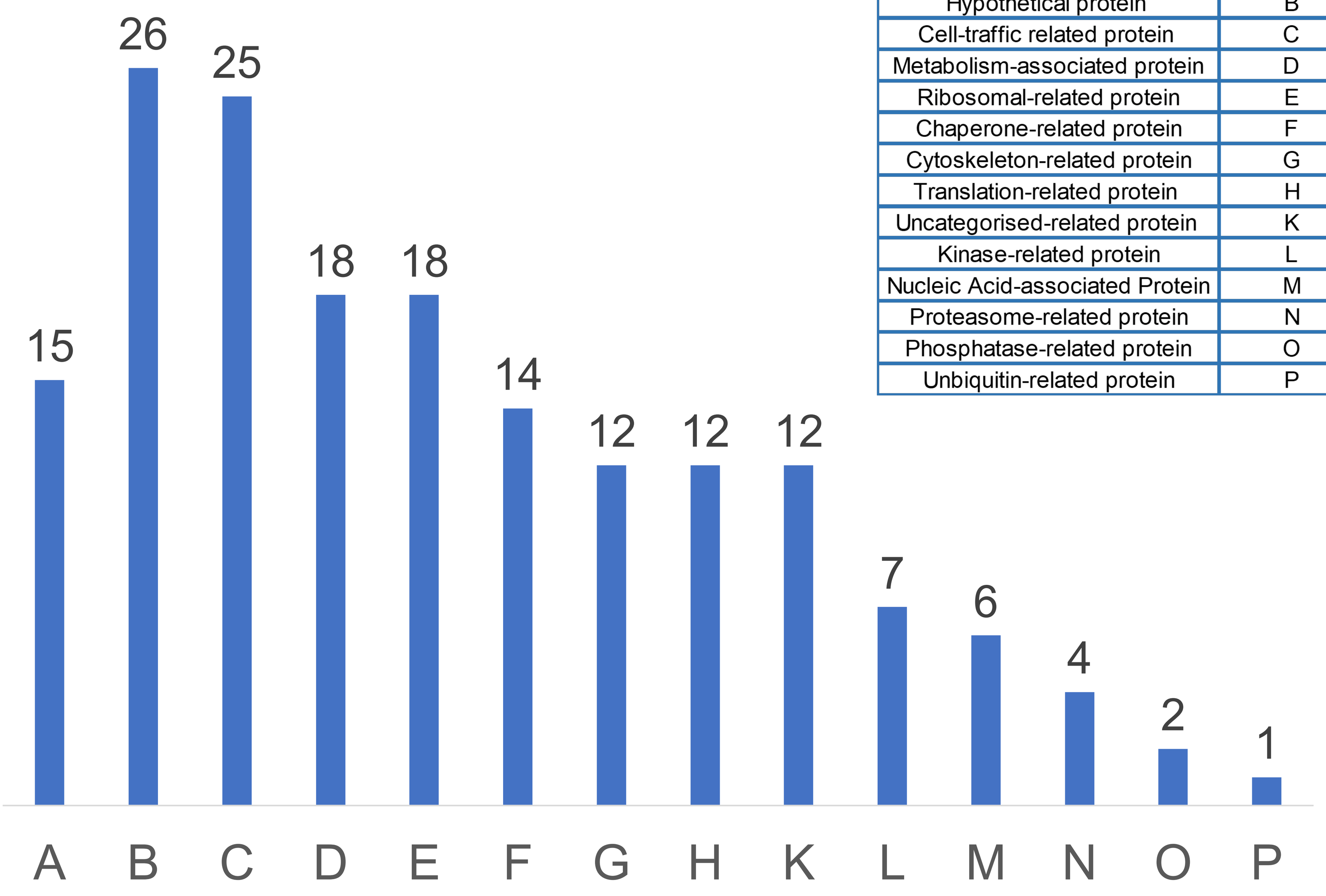

Protein Group	Graph code
Endocytic/Secretory pathway	A
Hypothetical protein	B
Cell-traffic related protein	C
Metabolism-associated protein	D
Ribosomal-related protein	E
Chaperone-related protein	F
Cytoskeleton-related protein	G
Translation-related protein	H
Uncategorised-related protein	K
Kinase-related protein	L
Nucleic Acid-associated Protein	M
Proteasome-related protein	N
Phosphatase-related protein	O
Unbiquitin-related protein	P

D

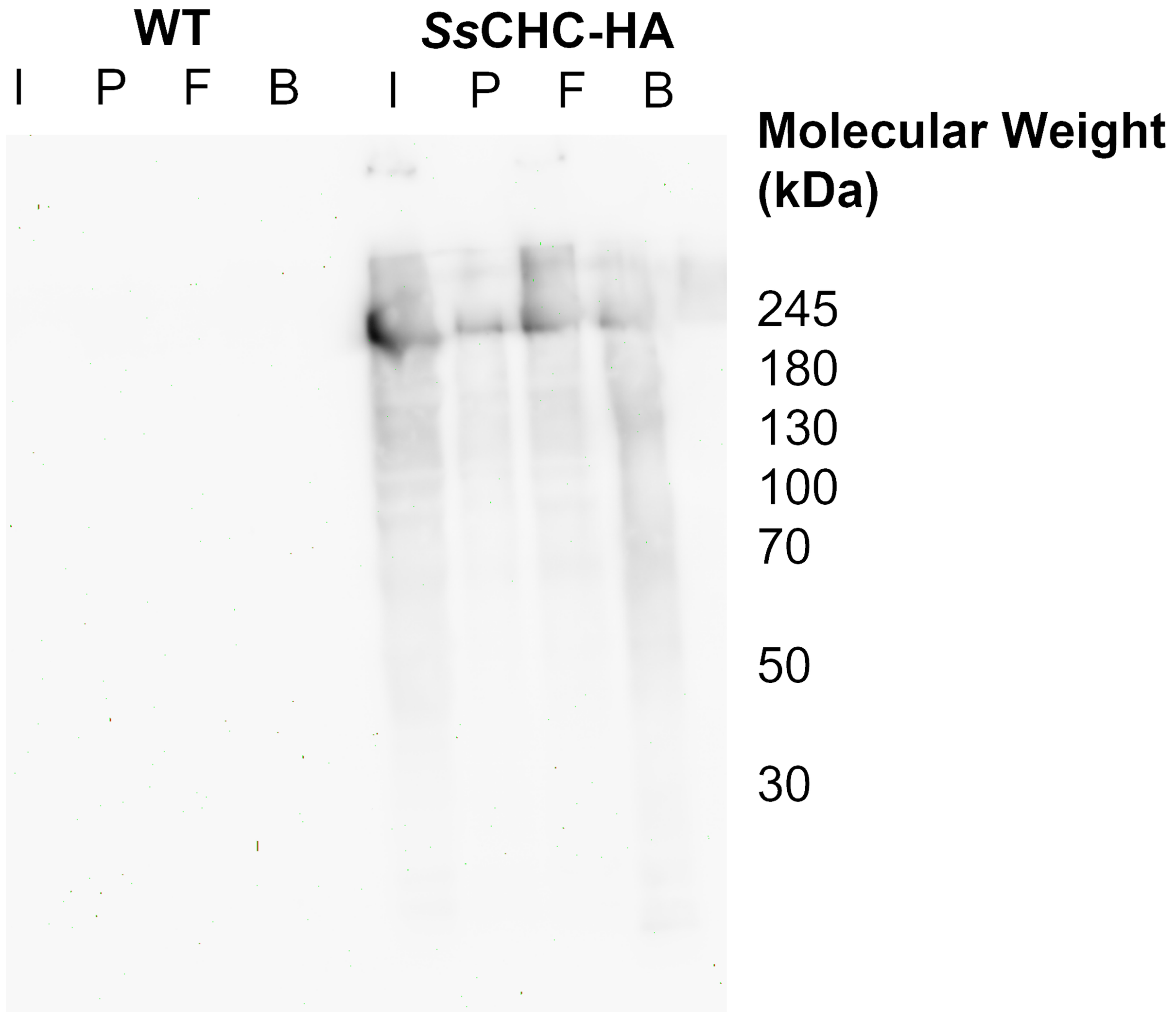

E

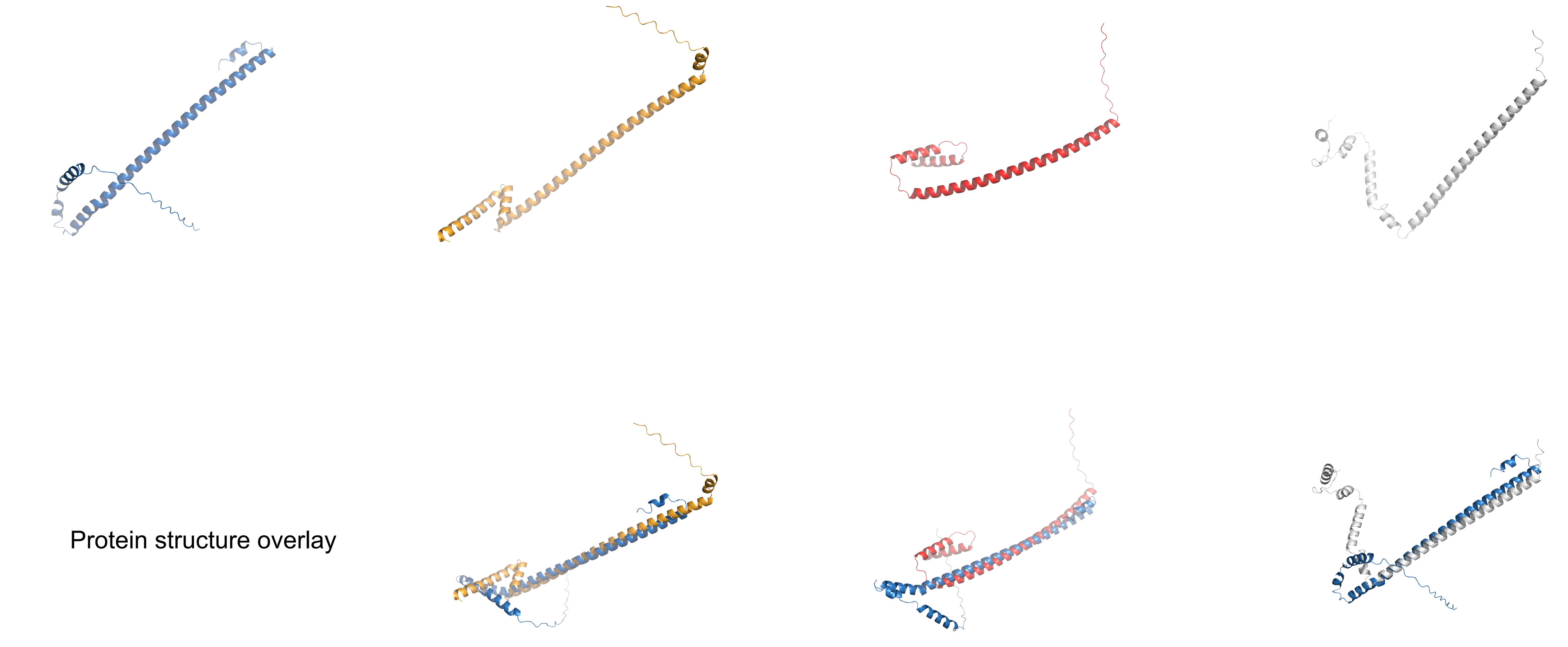

Specie/TM-align	*H. sapiens*	*T. brucei*	*G. lamblia*	*S. salmonicida*
H. sapiens	1	0.41651	0.48174	0.43935
T. brucei	0.41651	1	0.47487	0.56426
G. lamblia	0.48174	0.47487	1	0.55075
S. salmonicida	0.43935	0.56426	0.55075	1

Specie/RMSD (Å)	*H. sapiens*	*T. brucei*	*G. lamblia*	*S. salmonicida*
H. sapiens	0	4.58	4.58	5.32
T. brucei	4.58	0	6.17	8.54
G. lamblia	4.98	6.17	0	6.62
S. salmonicida	5.32	8.55	6.62	0
