# Supplementary material for: Combined nanometric and phylogenetic analysis of unique endocytic compartments in Giardia lamblia sheds light on the evolution of endocytosis in Metamonada
Source: BMC Biol. 2022 Sep 21;20:206. doi: 10.1186/s12915-022-01402-3 (PMC9490929; doi:10.1186/s12915-022-01402-3)
Supplement: Supplementary file 21 — Additional file 21: Fig. S10. Trepomonas sp. PC1 also harbours a putative CLC analogue. Ab initio protein modelling of TPC1_16039, orthologous to Ss11905 in combination with Ss11905, GlACLC, TbCLC and HsCLC. RMSD and TM-align cores show overall structural conservation with respect to a bona fide CLC. [file 12915_2022_1402_MOESM21_ESM.pdf]

Supplemental Figure 10

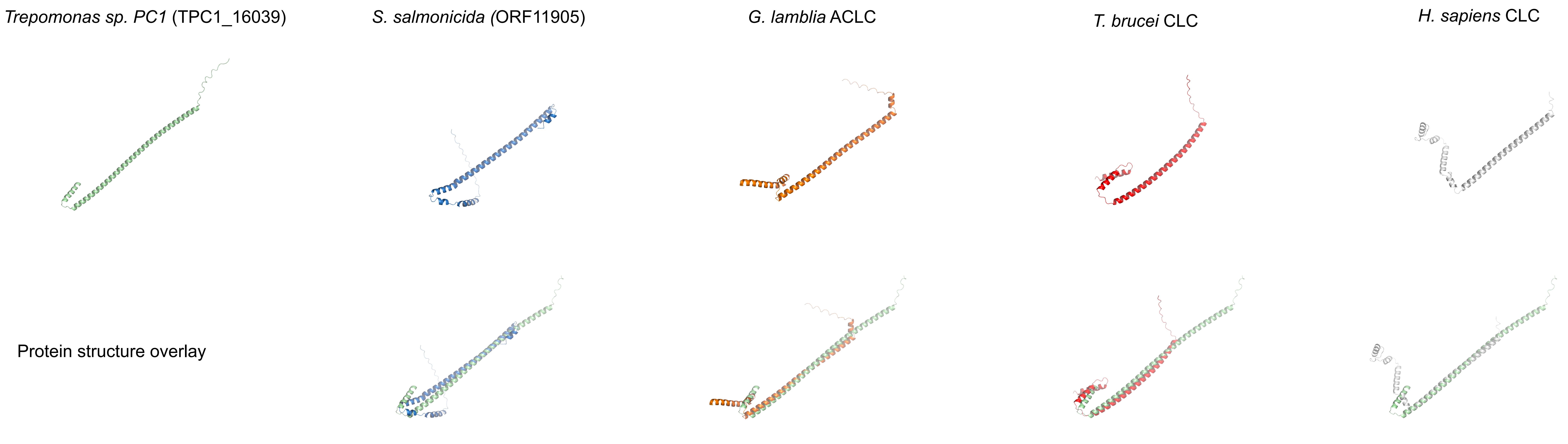

Specie/TM-align	*H. sapiens*	*T. brucei*	*G. lamblia*	*S. salmonicida*	*Trepomonas sp.* PC1
H. sapiens	1	0.41651	0.48174	0.43935	0.471
T. brucei	0.41651	1	0.47487	0.56426	0.46
G. lamblia	0.48174	0.47487	1	0.55075	0.56
S. salmonicida	0.43935	0.56426	0.55075	1	0.5
***Trepomonas sp.* PC1**	0.471	0.46	0.56	0.5	1

Specie/RMSD (Å)	*H. sapiens*	*T. brucei*	*G. lamblia*	*S. salmonicida*	*Trepomonas sp.* PC1
H. sapiens	0	4.58	4.58	5.32	3.1
T. brucei	4.58	0	6.17	8.54	4.69
G. lamblia	4.98	6.17	0	6.62	3.71
S. salmonicida	5.32	8.55	6.62	0	6.45
***Trepomonas sp.* PC1**	3.1	4.69	3.71	6.45	0
